# Use of Neuronal-Enriched Extracellular Vesicles to Distinguish Cognitive Impairment Levels and Sex Differences in People with HIV

**DOI:** 10.3390/cells15171581

**Published:** 2026-08-31

**Authors:** Norina Tang, Fan Xia, Heather Freasier, Phyllis C. Tien, Marshall J. Glesby, Daniel Merenstein, Audrey L. French, Heather McKay, Monica M. Diaz, Igho Ofotokun, Jordan E. Lake, Joseph B. Margolick, Eun-Young Kim, Steven R. Levine, Margaret A. Fischl, Wei Li, Jeremy Martinson, Lynn Pulliam

**Affiliations:** 1 Department of Laboratory Medicine, San Francisco VA Health Care System, San Francisco, CA 94121, USA; norina.tang@va.gov; 2 Department of Epidemiology and Biostatistics, University of California, San Francisco, CA 94158, USA; fan.xia@ucsf.edu; 3 Department of Medicine, University of California San Francisco, San Francisco, CA 94143, USA; heather.freasier@ucsf.edu (H.F.); phyllis.tien@ucsf.edu (P.C.T.); 4 Department of Medicine, San Francisco VA Health Care System, San Francisco, CA 94121, USA; 5 Department of Medicine, Weill Cornell Medical College, New York City, NY 10021, USA; mag2005@med.cornell.edu; 6 Department of Family Medicine, Georgetown University School of Medicine, Washington, DC 20007, USA; djm23@georgetown.edu; 7 Department of Medicine, Cook County Health, Chicago, IL 60612, USA; afrench@cookcountyhhs.org; 8 Department of Epidemiology, Johns Hopkins Bloomberg School of Public Health, Baltimore, MD 21205, USA; hmckay4@jhu.edu; 9 Department of Neurology, University of North Carolina at Chapel Hill School of Medicine, Chapel Hill, NC 27599, USA; monica.diaz@neurology.unc.edu; 10 Department of Medicine, Emory University School of Medicine, Atlanta, GA 30322, USA; iofotok@emory.edu; 11 Department of Internal Medicine, University of Texas Health Science Center at Houston, Houston, TX 77030, USA; jordan.e.lake@uth.tmc.edu; 12 Department of Molecular Microbiology and Immunology, Johns Hopkins Bloomberg School of Public Health, Baltimore, MD 21205, USA; jmargol1@jhu.edu; 13 Department of Medicine, Northwestern University Feinberg School of Medicine, Chicago, IL 60611, USA; e-kim@northwestern.edu; 14 Department of Neurology, Downstate Medical Sciences University, State University of New York College of Medicine, Brooklyn, NY 11203, USA; steven.levine@downstate.edu; 15 Department of Medicine, University of Miami, Miami, FL 33136, USA; mfischl@med.miami.edu; 16 Department of Clinical and Diagnostic Sciences, University of Alabama, Birmingham, AL 35294, USA; wli@uab.edu; 17 Department of Infectious Diseases and Microbiology, School of Public Health, University of Pittsburgh, Pittsburgh, PA 15260, USA; jmartins@pitt.edu; 18 Department of Laboratory Medicine, University of California, San Francisco, CA 94143, USA

**Keywords:** biomarkers, neuron-enriched extracellular vesicles, HIV-associated neurocognitive disorder (HAND), neuronal, extracellular vesicle, cognitive impairment, sex differences, GLRX, NCAM-1, pTau217

## Abstract

**Highlights:**

**What are the main findings?**

- Plasma neuronal-enriched extracellular vesicle (nEV) protein cargo in people with HIV (PWH) differed between the sexes.
- Within each sex, there were different patterns of nEV protein cargo that distinguished between the normal (NPN), asymptomatic (ANI), and mild (MND) cognitive groups in PWH.

**What are the implications of the main findings?**

- This exploratory study suggests it may be possible to use nEV protein markers to differentiate between NPN, ANI, and MND cognitive categories in PWH.

**Abstract:**

Cognitive impairment still occurs despite well-controlled HIV but with milder symptoms. People with and without HIV have different neuronal protein changes that may differentiate the asymptomatic neurocognitive impairment (ANI) condition from normal cognition (NPN) and mild neurocognitive disorder (MND). Plasma neuronal-enriched extracellular vesicles (nEVs) were isolated from people with HIV (PWH) with NPN, ANI, or MND, and HIV seronegative controls with NPN (HIV−). Two different platforms were used to delineate protein patterns between sexes and cognitive conditions. When combining results from men and women, the nEV cargo in many cases showed little difference between cognitive conditions. However, when separated, there were different patterns between the sexes for some nEV cargo that distinguished the HIV− groups and HIV+ cognition groups. PWH with NPN had higher nEV toxic proteins compared to individuals without HIV. Women with HIV showed perfect separation between NPN and ANI with Aβ42 and NCAM-1 and perfect separation between ANI and MND with Aβ40 and NCAM-1. Men with HIV with MND had significantly higher NCAM-1 when compared to ANI, and lower GLRX when compared to NPN. This study shows distinct markers for different HIV cognitive categories, many of which differed between men and women.

## 1. Introduction

In the current age of effective antiretroviral therapy (ART), HIV-associated neurocognitive disorder (HAND) has shifted from severe dementia to milder, persistent forms of cognitive impairment. Despite viral suppression to undetectable levels in the blood, around 20–50% of people with HIV (PWH) still experience cognitive impairment [1]. This may be a result of structural changes and alterations in neuronal activity since long-term ART users often show reduced brain volume and signs of neuronal injury and dysfunction compared to controls without HIV (HIV−) [2]. The mechanism behind HAND and brain changes is unclear and likely to be multi-factorial. Current testing for HAND relies on a two-pronged, resource-intensive approach involving clinical and molecular testing. Clinical testing entails clinical evaluation, neuropsychological (NP) testing and/or neuroimaging. These clinical tests are not only time-consuming and costly, but also the interpretation of the results can be subjective and inconsistent across different diagnostic/classification tools (e.g., International HIV Dementia Scale (IHDS), Montreal Cognitive Assessment (MoCA), Frascati Criteria). Molecular testing includes sampling of the cerebrospinal fluid (CSF) and/or blood to look for soluble markers of neuronal damage or signaling alterations, endothelial dysfunction, inflammation and/or immune cell changes [3,4]. While biomarkers in the CSF have been studied more frequently due to its proximity to the brain, lumbar puncture to obtain CSF is uncomfortable and invasive. In contrast, blood sampling by venipuncture is easy and well tolerated and, thus, more suitable for regular clinical monitoring. Furthermore, blood biomarkers are objective and quantifiable, possibly providing metrics to diagnose and monitor disease and treatment response. Despite these advantages, studies identifying potential biomarkers in the plasma/serum have been few and far between, with only a small number of potential markers being investigated [3,5,6,7]. Moreover, these limited studies have focused on markers of peripheral inflammation or neuronal cell death, which may not capture the more subtle changes in the brains of people with HAND who exhibit asymptomatic or mild cognitive impairment. To date, none of the molecular biomarkers identified thus far, whether CSF or blood, is specific to HAND. Moreover, it has been a struggle to identify milder cognitive function impairment levels such as ANI, which comprises 26% of the HAND cases in the current era of effective ART [8].

Extracellular vesicles (EVs) are small lipid bilayer structures secreted by cells, which carry biological cargo reflective of the host cell, such as nucleic acids, lipids, and proteins. EVs secreted by a host cell can readily enter a recipient cell nearby or at a distance, thereby allowing them to act as key mediators of cellular communication and play crucial roles in HIV neuropathology [9,10,11]. EVs can also cross the blood–brain barrier, acting as a bridge between the central nervous system (CNS) and peripheral tissues [12]. Therefore, querying the biological cargo of EVs in the blood that are secreted from CNS cells may provide a window into CNS changes occurring due to HAND and yield biomarkers that can potentially diagnose or enhance diagnosis, especially for the less severe forms of cognitive impairment.

Our group has utilized and published findings on using L1CAM (L1 cell adhesion molecule, CD171) antibodies to affinity purify neuron-enriched EVs (nEVs) from the plasma in PWH and found that nEV cargo proteins differed between PWH who are neuropsychologically normal (NPN) and PWH who are neuropsychologically impaired [13]. Our previous studies utilized very small sample sizes from different cohorts that were not age-matched or sex-matched. To better address the limitations of the previous studies, we sought, in this study, to examine the plasma nEVs from a larger, more balanced cohort to determine whether there are differences between the two less severe forms of neurological disorder by NP categorization: ANI and MND. In addition, using two different panels of nEV neurodegenerative protein markers, we sought to determine whether there are sex differences between men with HIV (MWH) and women with HIV (WWH) on stable ART with undetectable viral load. In general, when the sexes were analyzed together, people without HIV (PWoH) had a trend toward lower levels of nEV neurodegenerative proteins compared to PWH with NPN, suggesting a neuroinflammatory response as a result of HIV infection and an increased secretion of accumulated toxic proteins. In addition, PWoH had significant differences in seven nEV proteins, with men having higher levels. Amongst PWH, we found six nEV protein markers that were lower in the ANI group than in NPN or MND groups. nEV protein markers were also lower in women than in men within the same neuropsychological category. Surprisingly, the nEV cargo levels in the HIV− and ANI groups were similar, despite the ANI group being HIV+ and having some deficiencies on NP testing. Also, nEV cargo levels were similar in PWH amongst the NPN and MND groups despite the latter showing deficiencies on NP testing along with noticeable clinical symptoms. Two nEV proteins, NCAM-1 (neural cell adhesion molecule 1) and GLRX (glutaredoxin), were distributed differently amongst the three cognition groups (NPN, ANI, MND) in MWH. In WWH, NCAM-1 showed perfect separation between NPN, ANI, and MND cognition groups. The results from this exploratory study suggest that protein markers in plasma nEVs may be utilized to enhance diagnosis of the categories of HAND and highlight the sex differences to consider.

## 2. Materials and Methods

### 2.1. Study Participants and Plasma Collection

A total of 140 age-matched plasma samples were selected from the MACS/WIHS Combined Cohort Study (MWCCS). Participant recruitment, characteristics, and NP testing information have been published previously [14,15,16]. People with co-morbidities were excluded. Of the 140 total plasma samples, 31 were from healthy HIV seronegative controls with normal cognition, 40 from PWH with NPN, 30 from PWH with ANI, and 39 from PWH with MND. Controls were evaluated with the same cognitive testing as the HIV+ cohort and assessed as normal. ANI is defined as impaired cognitive function based on at least two ability domains, with at least one standard deviation below the mean for normal age-matched people on neuropsychological tests. Further defining requirements are that there is no other pre-existing condition to explain this, and the deficit *does not interfere* with normal daily functioning. In contrast, MND has the same definition as ANI *except* that the neurocognitive deficit *does interfere* with normal daily functioning.

### 2.2. nEV Isolation

nEVs were purified using a two-step method as previously described [17] with slight modifications. The first step entails purifying total extracellular vesicles using the SmartSEC™ HT EV Isolation System (System Biosciences, Palo Alto, CA, USA; catalog #SSEC096A-1), which is a proprietary chromatography-based EV isolation method that comes in a 96-well format that combines size exclusion with a contaminant trapping feature to remove carryover proteins. The second step entails purifying nEV using L1CAM antibodies for selective affinity capture.

Briefly, step 1 of the nEV isolation procedure is as follows: 0.5 mL of frozen plasma was thawed and centrifuged at 3000× *g* for 15 min at room temperature (RT) to remove particulates. Following manufacturer’s instructions, 0.5 mL of clarified plasma from each sample was loaded into the corresponding well of the SmartSEC™ HT EV Isolation System 96-well plate. Total extracellular vesicles were eluted as two fractions, pooled, and stored at −80 °C until use in step 2 of the nEV isolation procedure.

In step 2 of the nEV isolation procedure, 4 μg of biotinylated L1CAM mouse monoclonal antibody (CD171, clone 5G3, ThermoFisher Scientific, Rockford, IL, USA, catalog #13-1719-32) in 3% bovine serum albumin (BSA; Amresco, LLC, Solon, OH, USA; product code K719) was added to each sample of thawed total EVs and incubated for 3 h at RT; after which, 80 μL of streptavidin-conjugated agarose beads (Pierce™ Streptavidin Plus UltraLink™ Resin (Thermo-Fisher Scientific, catalog #53117) in 3% BSA was added and the mixture incubated for 1.5 h at 4 °C. The L1CAM+ EV bead complexes generated above were then washed with phosphate-buffered saline (PBS; Thermo-Fisher Scientific, catalog #14190144) and 200 μL of 0.1 M Glycine-HCl, pH 3.0 (Thermo-Fisher Scientific, catalog #J62527.AK) added for 10 s on ice, followed by centrifugation at 4500× *g* for 5 min at 4 °C. The supernatants containing the acid-released nEV were quickly collected, neutralized with 30 μL of 1 M Tris-HCl, pH 8 (Thermo-Fisher Scientific, catalog #J22638.AE), aliquoted, and stored at −80 °C until analysis.

### 2.3. nEV Lysate Preparation

Immediately after purifying the nEVs as stated above, 100 μL of it was used to generate nEV lysates by adding 200 μL of Mammalian Protein Extraction Reagent (M-PER, ThermoFisher Scientific, catalog #78501) supplemented with BSA (0.25% final concentration) and HALT™ protease and phosphatase inhibitors (2× final concentration) (ThermoFisher Scientific, catalog #78441). Lysed nEVs were subjected to two freeze–thaw cycles at −80 °C, aliquoted, and stored at −80 °C until use.

### 2.4. Nanoparticle Tracking Analysis (NTA)

Purified nEV samples were analyzed by NTA using a ZetaView^®^ ×20 QUATT instrument (Particle Metrix GmbH, Inning am Ammersee, Germany) equipped with a 488 nm laser chamber and ZetaView^®^ version 8.06.01 SP1 software to determine size (median and mean) and total particle number (counts). Prior to data acquisition, instrument calibration was performed using a known concentration of 100 nm polystyrene beads supplied by the equipment manufacturer. For each nEV sample, triplicate readings were obtained. Each reading consisted of 11 positions with 2 cycles for each position at a sensitivity of 75, frame rate of 60, and a shutter value of 100. Reported values represent averages of the triplicate readings. The investigator was blinded to the identity of the samples.

### 2.5. Electrochemiluminescent Multiplex Assay for Tetraspanin Proteins

A custom electrochemiluminescent multiplex immunoassay kit from Meso Scale Discovery (MSD; Rockville, MD, USA; catalog #K15228N-1) was used to quantify the protein levels of CD9, CD63, and CD81 in the isolated nEVs using antibody set catalog numbers F215M-3, F215L-3, and F215N-3, respectively. Each sample was tested in duplicate following the manufacturer’s supplied protocol and results read using a QuickPlex SQ 120 instrument (MSD). Quantification analyses were performed using DISCOVERY WORKBENCH^®^ 4.0 software (MSD). All reported values were normalized to plasma volume. Sample identities were blinded to the investigator.

### 2.6. Fluorescent Multiplex Assays for Plasma and nEV Proteins

Two custom magnetic, bead-based, multiplex immunoassays utilizing Luminex xMAP™ technology were used to quantify the proteins of interest in the nEVs: 5-plex and 10-plex. Both were Invitrogen ProcartaPlex assays purchased from ThermoFisher Scientific.

Procarta 5-plex (SKU #PPX-05-MXZTGXT) was used to quantify GAPDH, HSP90, S100B, αSyn (total), and NFL in the nEV lysates. Lower limits of detection for the 5-plex proteins were as follows: GAPDH, 6.05 pg/mL; HSP90, 5.92 pg/mL; S100B, 0.01 pg/mL; αSyn, 12.99 pg/mL; NFL, 7.86 pg/mL.

Procarta 10-plex (SKU #PPX-10-MXDJZ65) was used to quantify Aβ40, Aβ42, FGF-21, HMGB1, KLK-6; NCAM-1, NRGN, pTau181, TDP-43, and tTau in the nEV lysates. Lower limits of detection for the 10-plex proteins were as follows: Aβ40, 72.13 pg/mL; Aβ42, 0.19 pg/mL; FGF-21, 2.41 pg/mL; HMGB1, 128.52 pg/mL; KLK-6, 0.67 pg/mL; NCAM-1, 31.74; NRGN, 6.28; pTau181, 0.39 pg/mL; TDP-43, 460.08 pg/mL; tTau 16.31 pg/mL.

All Luminex bead assays were performed in duplicate following manufacturer’s instructions. Results were read using a FLEXMAP 3D instrument with Luminex xMAP™ Technology. Data were analyzed using Milliplex^®^ Analyst version 5.1 software. All reported values were normalized to plasma volume. All Luminex assays were performed blinded.

### 2.7. Proximity Extension Assay (Olink^®^) for Neurodegenerative Proteins

Proximity extension assays were performed on the nEV lysates in singlet per manufacturer’s instructions at the University of California, San Francisco Core Immunology Lab (San Francisco, CA, USA) using the Olink^®^ Target 48 Neurodegeneration multiplex assay kit (Olink^®^ Proteomics Inc., Waltham, MA, USA; product #s 93600 and 93007). Data acquisition and analysis were conducted using the Olink^®^ Signature Q100 instrument with Olink^®^ Signature Q100 software (version 2.0.0.57), compliant with 21 CFR Part 11 requirements. Sample identities were concealed from the investigators until data analysis was complete. Results were reported as normalized protein expression (NPX) values. NPX is Olink’s relative quantification unit, expressed on a log2 scale. NPX measures relative change. A higher NPX translates to a higher protein abundance in the sample.

Although the Olink^®^ Target 48 Neurodegeneration multiplex assay also provided quantitative values, we did not report or use these quantitative values for analyses because some were below detection. Nevertheless, we showed the quantitative values for GLRX and NPTX2 (Figure A4) because these were all detectable and, thus, were used for multiple logistic regression analyses (Table A1).

### 2.8. Statistical Analyses

GraphPad Prism 10 software (GraphPad Software, Inc., Boston, MA, USA) was used in all statistical analyses. In the figures, * denotes *p* ≤ 0.05, ** denotes *p* ≤ 0.01, *** denotes *p* ≤ 0.001, and **** denotes *p* ≤ 0.0001. These *p*-values are calculated using a one-way ANOVA test, with a post hoc Tukey test to correct for multiple comparisons used when the data were normally distributed. When the data were not normally distributed, the Kruskal–Wallis test with post hoc Dunn’s test to correct for multiple comparisons was used when comparing group differences. Categorical group differences were assessed using Fisher’s exact test. Correlations between quantitative variables were determined using Spearman’s two-tailed test with simple linear regression to illustrate trend lines. Multivariate logistic regression analyses using the main effects model were performed to assess the ability of predictors to discriminate ANI cognitive status.

## 3. Results

### 3.1. Participant Demographic and Clinical Data

The clinical samples were selected from participants in the MACS/WIHS Combined Cohort Study whose demographic characteristics are shown in Table 1. Of the 140 plasma samples used to isolate nEVs, 72 (51%) were men and 68 (49%) were women. These were further divided into 4 cognitive categories: 31 participants without HIV (PWoH) with normal neurocognition (HIV−), 40 PWH who tested NPN, 30 PWH with ANI, and 39 PWH with MND. All PWH had undetectable viral loads of <20 copies/mL of blood. The mean age of men was 52 years, with a range of 44–66 years. For women, the mean age was also 52 years, with a range of 45–65 years. There was a wide range for years on ART in both sexes, with a mean of roughly 10 years for both. The cohort was predominantly black (72%) with similar distribution between cognition categories. There was little difference in education between sexes, and most had graduated from high school with some college.

### 3.2. nEV Characterization

**Figure A1:**
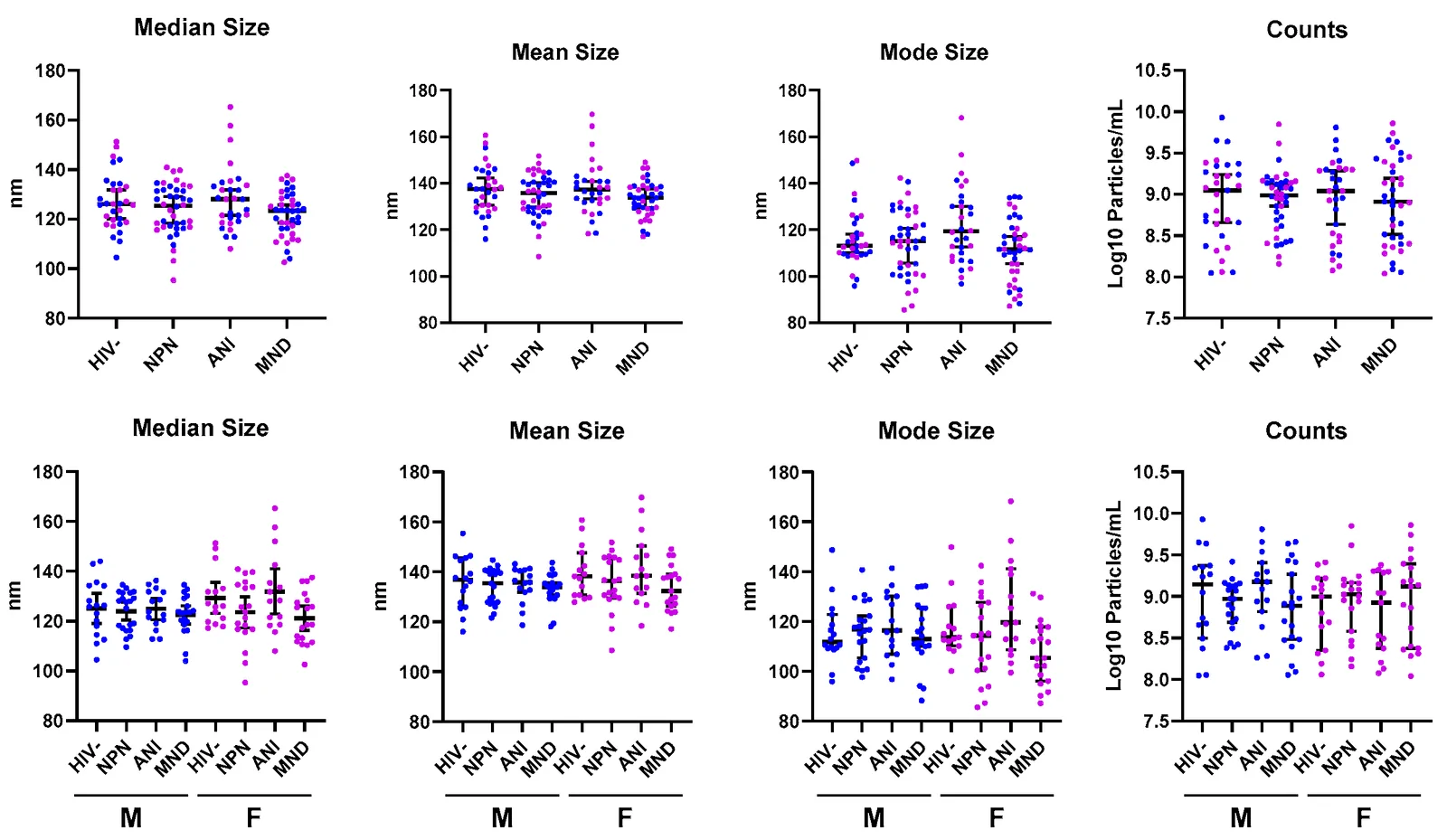
Nanoparticle tracking analysis (NTA). There were no differences in nEV size or concentration between cognitive categories or sex. Males are blue circles and females are pink circles. Sample sizes for males (M), HIV− (*n* = 16), NPN (*n* = 21), ANI (*n* = 15), and MND (*n* = 20). For females (F), the sample sizes were HIV− (*n* = 15), NPN (*n* = 19), ANI (*n* = 15), and MND (*n* = 19). Horizontal bars indicate group medians and 95% confidence intervals as determined using Kruskal–Wallis tests, followed by post hoc Dunn tests to correct for multiple comparisons.

**Figure A2:**
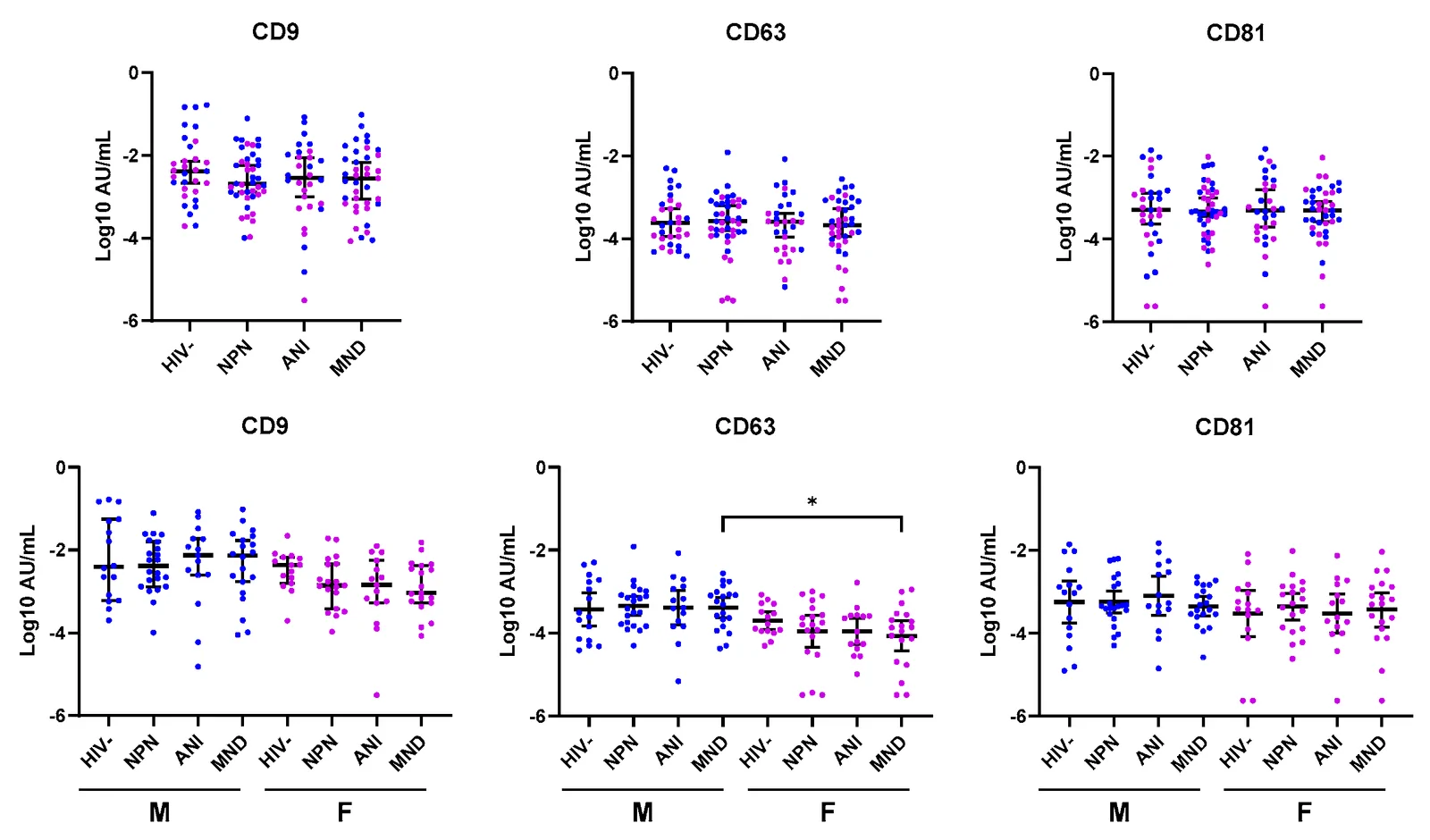
Tetraspanins on nEVs. MSD was used to quantify CD9, CD63, and CD81 on nEVs. Only CD63 showed a difference, with a higher concentration of nEVs from males (M) with MND compared to females (F). Sample sizes for males (blue circles), HIV−(*n* = 16), NPN (n = 21), ANI (*n* = 15), and MND (*n* = 20). For females (pink circles), the sample sizes are HIV− (*n* = 15), NPN (*n* = 19), ANI (*n* = 15), and MND (*n* = 19). Horizontal bars indicate group medians with 95% confidence intervals as determined using Kruskal–Wallis tests, followed by post hoc Dunn tests for data that are not normally distributed (CD9 top and bottom graphs, CD63 top graph, CD81 top graph). For data that are normally distributed (CD63 bottom graph, CD81 bottom graph), horizontal bars represent group means and 95% confidence intervals as determined by ordinary one-way ANOVA followed by Tukey tests to correct for multiple comparisons. Only one group difference was found and is shown with an asterisk defined as * *p* ≤ 0.05.

nEVs were isolated from frozen plasma using chromatography followed by affinity capture with biotinylated L1CAM monoclonal antibodies to enrich for neuronal EVs [17]. In all analyses on nEVs, we first combined men and women and compared the 4 sample groups (HIV−, NPN, ANI, MND) to determine if nEV proteins could differentiate between the groups. We then separated the men and women in each sample group to see if there were sex differences and whether or not one sex was driving a particular group difference. nEV characterization included NTA for size and concentration (Figure A1). Most of the nEVs were in the range of 100–140 nm, the size of exosomes. There were no differences in size or concentration of nEVs separating men and women or between the 4 sample groups. Three tetraspanins, CD9, CD63, and CD81, were used to characterize the nEVs using an MSD multiplex platform. WWH with MND had lower levels of CD63 in nEVs compared to MWH with MND (Figure A2). This may imply that the inflammatory response is stronger in MND for MWH than for WWH since CD63 participates in endothelial cell activation and initiation of inflammation by helping to recruit leukocytes to the endothelium [18].

**Figure 1:**
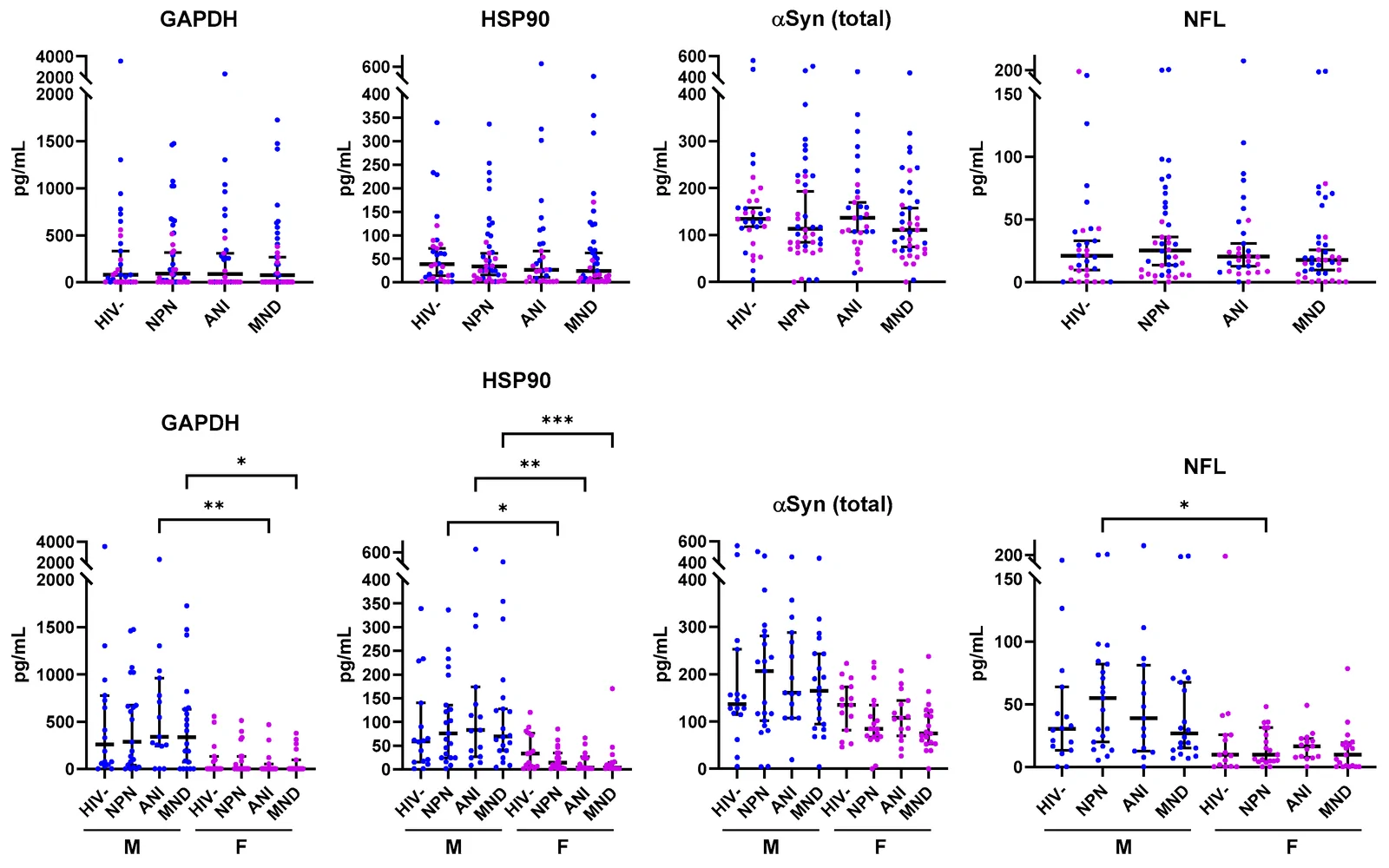
nEV characterization. GAPDH and HSP90 are extracellular vesicle proteins and αSyn and NFL are neuronal proteins. The top row combines the sexes into 4 sample groups. Males are blue circles and females are pink circles. The bottom row differentiates males (M) from females (F), showing higher protein levels in men. Sample sizes are as indicated in Table 1. Horizontal bars indicate group medians and 95% confidence intervals as determined using Kruskal–Wallis tests, followed by post hoc Dunn tests to correct for multiple comparisons. Group differences are shown with asterisks defined as follows: * *p* ≤ 0.05, ** *p* ≤ 0.01, and *** *p* ≤ 0.001.

Further characterization of nEVs was performed using a Luminex 5-plex panel on the lysed vesicles (Figure 1). Although the 5-plex panel included S100B (S100 calcium binding protein B), a protein present primarily in astrocytes, results for S100B protein levels were excluded from Figure 1 since most values were below detection. When the men and women were grouped together (top row, Figure 1), there were few differences between the four sample groups. However, when separated, as shown in the bottom row of Figure 1, MWH with ANI had higher levels of cytoplasmic proteins, GAPDH (glyceraldehyde-3-phosphate dehydrogenase), and HSP90 (heat shock protein 90) than WWH with ANI. The same was seen for the MND groups, where MWH with MND showed higher levels of GAPDH and HSP90 than WWH with MND. HSP90 was also higher in MWH with NPN compared to WWH with NPN. αSyn (alpha-synuclein) and NFL (neurofilament light polypeptide), both neuronal proteins, were similar across all sample groups, with men generally having higher nEV levels than women.

### 3.3. nEV Protein Cargo Analyses Using Luminex Fluorescent Multiplex Assay

**Figure 2:**
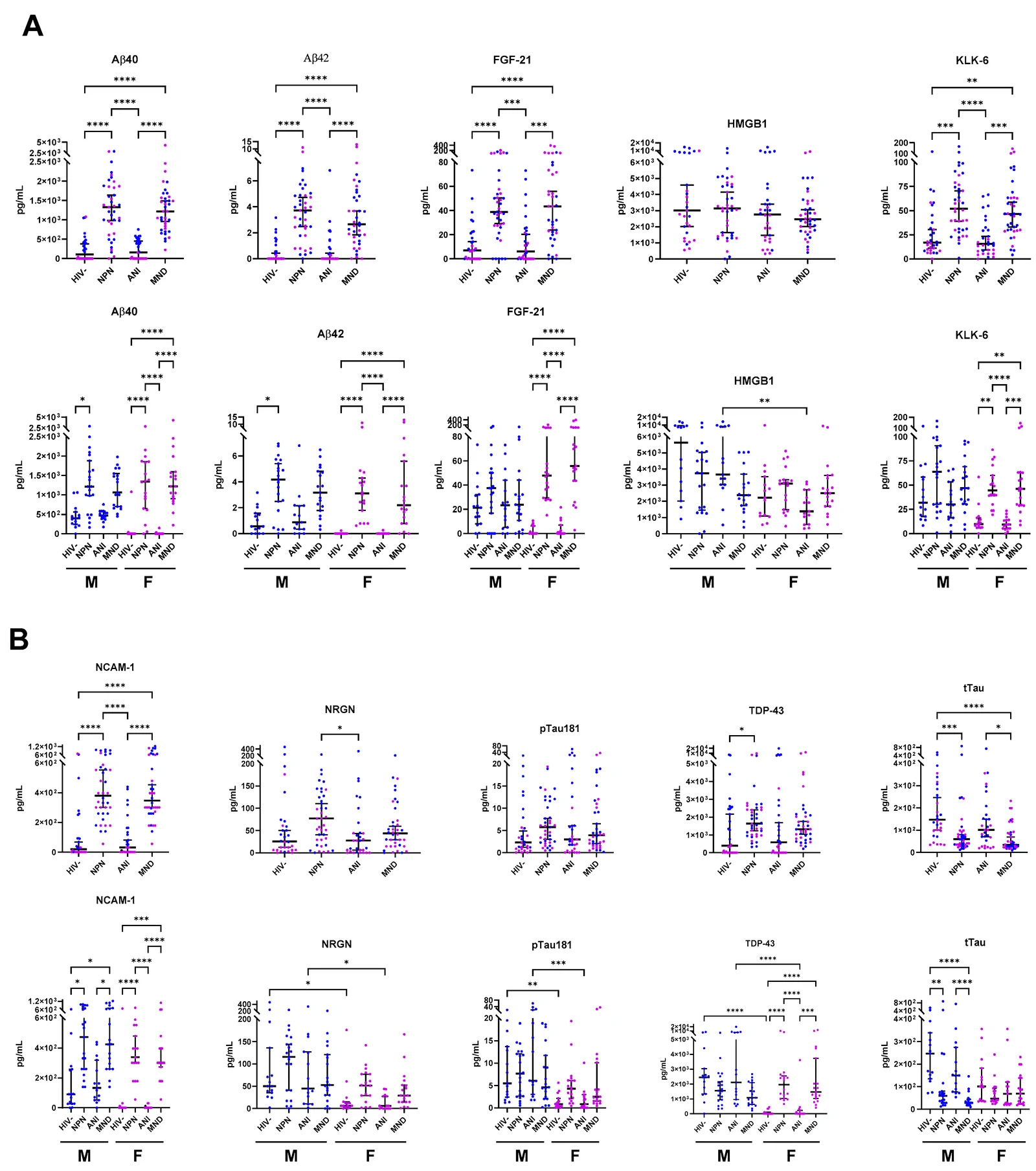
nEV 10-plex protein cargo (Luminex). (**A**,**B**) Top row: males and females combined into 4 sample groups. Males are blue circles and females are pink circles. Bottom row: males (M) and females (F) separated into the 4 cognition groups, HIV−, NPN, ANI, and MND. Due to sample limitations, sample numbers were slightly different between the groups. Sample sizes for males: HIV− (*n* = 15), NPN (*n* = 21), ANI (*n* = 15) and MND (*n* = 19). For females, the sample sizes were HIV− (*n* = 15), NPN (*n* = 18), ANI (*n* = 15) and MND (*n* = 19). Horizontal bars indicate group medians and 95% confidence intervals as determined using Kruskal–Wallis tests, followed by post hoc Dunn tests to correct for multiple comparisons. Group differences are shown with asterisks defined as follows: * *p* ≤ 0.05, ** *p* ≤ 0.01, *** *p* ≤ 0.001, and **** *p* ≤ 0.0001.

Isolated nEVs allow us to query neuron health at the time of isolation and, in the case of cognitive impairment, what toxic products are secreted from neurons into the brain and periphery. We used a Luminex 10-plex neurodegeneration panel that tests well-established toxic proteins associated with cognitive impairment. When men and women were analyzed together (Figure 2A, top row), Aβ40 (amyloid-beta precursor protein 40) and Aβ42 (amyloid-beta precursor protein 42) were higher in PWH with NPN and MND than in PWoH. Aβ40 and Aβ42 were also higher in the NPN and MND sample groups when compared to ANI. When the sexes were separated, it was the women who showed visible differences between the sample groups. In WWH, there were significant differences in Aβ40 and Aβ42 peptide levels when comparing HIV− to NPN, HIV− to MND, NPN to ANI, and ANI to MND. Although MWH with NPN had higher Aβ peptides compared to MWoH (Figure 2A, bottom row), there were no differences amongst the three cognition groups in MWH. For Aβ40 and Aβ42, it appears that differences between sample groups when men and women were combined were primarily driven by women.

The pattern for Aβ40 and Aβ42 was also largely observed for FGF-21 (fibroblast growth factor 21), KLK-6 (kallikrein-related peptidase 6), and NCAM-1, with minor differences. For FGF-21, KLK-6, and NCAM-1, when men and women were combined into the four sample groups (Figure 2A,B, top rows), a pattern identical to that of the Aβ peptides was observed. When the sexes were separated, women showed the same pattern for FGF-21, KLK-6, and NCAM-1 as they did for the Aβ peptides. For men, the differences between HIV− and NPN, observed for the Aβ peptides, were no longer present for FGF-21 and KLK-6. NCAM-1 in men not only showed the same group differences as the Aβ peptides, but also additional differences between HIV− and MND, and between ANI and MND.

HMGB1 (high mobility box 1) and pTau181 (phosphorylated tau at threonine 181) showed no significant differences between the groups when men and women were combined. When the sexes were separated, MWH with ANI had higher levels of HMGB1 than WWH with ANI. Sex differences were also observed for pTau181, where men exhibited higher protein levels than women for the HIV− and ANI groups.

For NRGN (neurogranin), TDP-43 (TAR DNA-binding protein 43), and tTau (total tau), group differences were observed when the sexes were combined as well as when the sexes were separated. When the sexes were combined, NRGN showed differences between NPN and ANI; TDP-43 showed differences between HIV− and NPN; and tTau showed differences between HIV− and NPN, between HIV− and MND, and between ANI and MND (Figure 2B, top row). When the sexes were separated, nEVs from MWoH were higher in NRGN and TDP-43 (TAR DNA-binding protein 43) than in WWoH (Figure 2B, bottom row). NRGN was also lower in both WWH with ANI, compared to MWH with ANI. Looking at TDP-43, women showed a visible separation between the 4 groups that was absent in the men (Figure 2B, bottom row). In contrast to TDP-43, for tTau, men (not women) showed a visible separation between the 4 groups. In summary, when men and women were combined, there were visible differences between groups for Aβ40, Aβ42, FGF-21, KLK-6, NCAM-1, and TDP-43, with protein levels being higher in NPN and MND than HIV− and ANI sample groups. When separated into men and women, it was the women driving the significant differences between HIV−, NPN, ANI, and MND for these six markers. In contrast to these 6 protein markers, for tTau, it appears that men, and not women, were the driving force behind the group differences when the sexes were combined.

### 3.4. Discriminating Between Sex and Between PWH Cognition Groups

**Figure 3:**
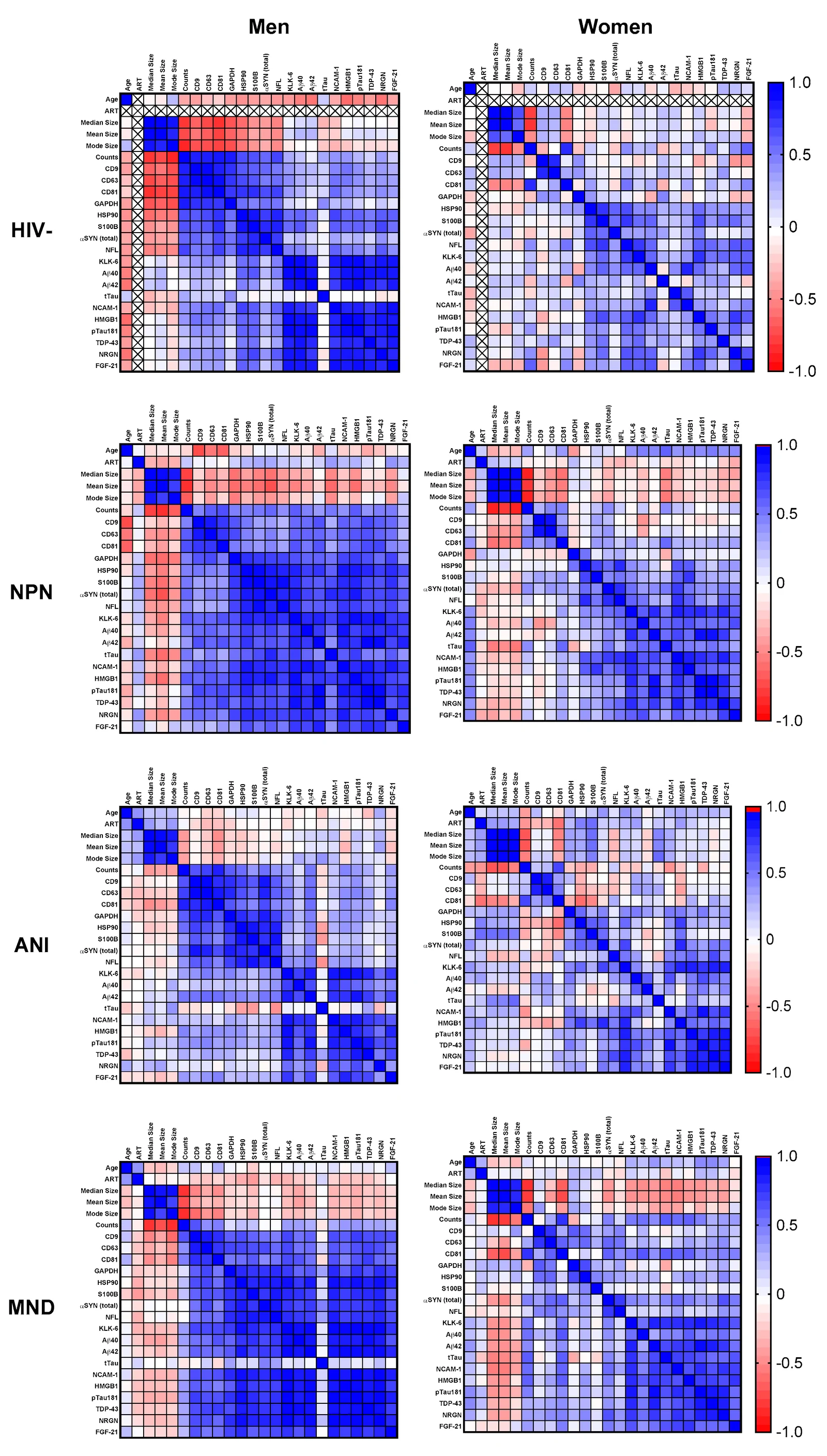
Multi-analyte correlation matrices between sample groups (HIV−, NPN, ANI, and MND) and sex using Spearman rank correlation. Positive correlations are in blue and negative correlations in red, with the extent demonstrated in the deeper color as shown in the bar to the right. Due to availability of clinical information, sample limitation, and assay sensitivity, sample sizes varied depending on the group and marker. For the HIV− group: Age, *n* = 22 men and 18 women; nEV Median Size, Mean Size, Mode Size, Counts, CD9, CD63, CD81, GAPDH, HSP90, αSyn, and NFL, *n* = 16 men and 15 women; KLK-6, Aβ40, Aβ42, tTau, NCAM-1, HMGB1, pTau181, TDP-43, NRGN and FGF-21, *n* = 15 men and 15 women. For the NPN group: Age, *n* = 23 men and 22 women; ART, *n* = 22 men and 22 women; nEV Median Size, Mean Size, Mode Size, Counts, CD9, CD63, CD81, GAPDH, HSP90, αSyn, and NFL, *n* = 21 men and 19 women; KLK-6, Aβ40, Aβ42, tTau, NCAM-1, HMGB1, pTau181, TDP-43, NRGN and FGF-21, *n* = 21 men and 18 women. For the ANI group: Age, *n* = 19 men and 17 women; ART, *n* = 19 men and 17 men; nEV Median Size, Mean Size, Mode Size, Counts, CD9, CD63, CD81, GAPDH, HSP90, αSyn, and NFL, *n* = 15 men and 15 women; KLK-6, Aβ40, Aβ42, tTau, NCAM-1, HMGB1, pTau181, TDP-43, NRGN and FGF-21, *n* = 15 men and 15 women. For the MND group: Age, *n* = 21 men and 21 women; ART, *n* = 21 men and 21 women; nEV Median Size, Mean Size, Mode Size, Counts, CD9, CD63, CD81, GAPDH, HSP90, αSyn, and NFL, *n* = 20 men and 19 women; KLK-6, Aβ40, Aβ42, tTau, NCAM-1, HMGB1, pTau181, TDP-43, NRGN and FGF-21, *n* = 19 men and 19 women.

By combining age, years on ART, and the various nEV protein cargo, we were able to construct correlation matrices for men and women in each sample group: HIV−, NPN, ANI, and MND (Figure 3). Values with a positive Spearman correlation of > 0 are blue; those with a negative correlation < 0 are red. Looking at these matrices, one can easily see differences between the sample groups and between the sexes. The correlation matrices show that men have more positive correlations in NPN and MND, unlike the women. Men also tend to have stronger correlations among the 4 groups and when compared to women.

To more clearly identify potential biomarkers to discriminate between the NP groups and between the sexes in PWH, we performed logistic regression with the nEV proteins along with age and years on ART using the main effects model (Table 2). The results are divided into two main sections: the top half shows single predictor variables for discrimination between NPN and ANI cognition categories, while the bottom half of the table shows single predictor variables for discrimination between ANI and MND cognition categories. Results for males are shown on the left and results for females are shown on the right. Predictor variables are listed based on the area under the curve (AUC) from highest to lowest. The regression results showed that, in men, the top single predictor biomarkers to discriminate between NPN and ANI, and between ANI and MND, were similar and consisted of NCAM-1, Aβ40, and tTau. In men, NCAM-1 and Aβ40 both had AUCs above 0.80 when discriminating between NPN and ANI. tTau, Aβ40, and NCAM-1 all had AUCs above 0.80 when discriminating between ANI and MND in men.

In contrast, when discriminating between NPN and ANI in women, the top single predictors with AUCs above 0.80 were Aβ42, NCAM-1, FGF-21, KLK-6, Aβ40, TDP-43, pTau181, and NRGN. When discriminating between ANI and MND in females, the top predictors were Aβ40, NCAM-1, FGF-21, TDP-43, KLK-6, and Aβ42.

Within each sex, the cluster of top predictors was similar when discriminating between the NP groups. However, the protein clusters differed between males and females, suggesting possible different mechanisms of cognitive impairment between the sexes.

**Figure 4:**
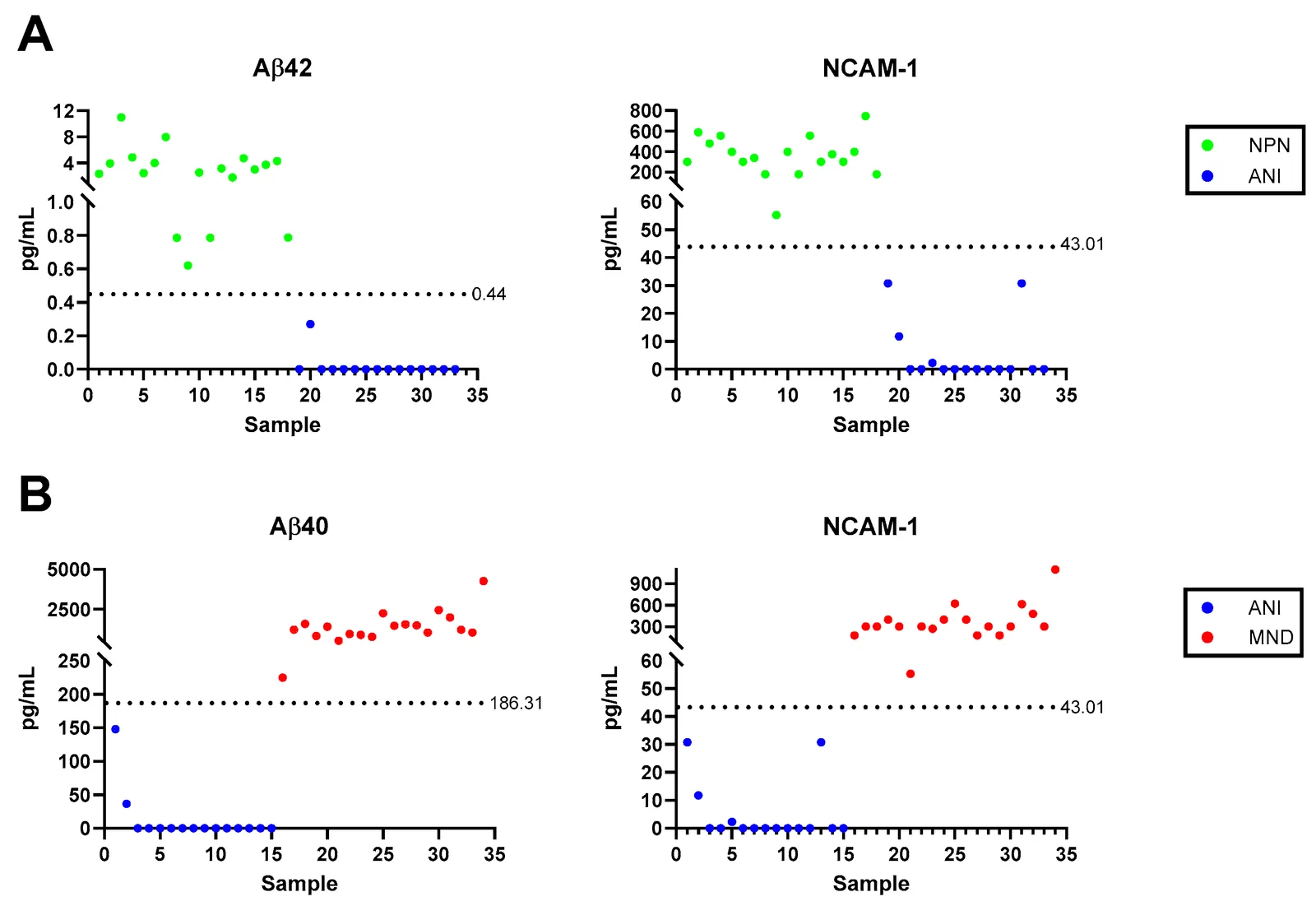
Scatter plots showing perfect separations in females for several single predictors. (**A**) Discrimination between NPN (*n* = 18, green circles) and ANI (*n* = 15, blue circles) NP groups. (**B**) Discrimination between ANI (*n* = 15, blue circles) and MND (*n* = 19, red circles) NP groups. Dotted lines represent the halfway point between the minimum value of the first group and the maximum value of the second group.

A closer look at the logistic regression analyses (Table 2) and protein expression levels (Figure 2) showed that in women, there was perfect separation between NPN and ANI categories of cognition (Figure 4A). In NPN, all women had nEV concentrations of Aβ42 above 0.44 pg/mL and NCAM-1 concentrations above 43.01 pg/mL. In ANI, all women had Aβ42 levels below 0.44 pg/mL plasma and NCAM-1 levels below 43.01 pg/mL plasma. There was also perfect separation in women between ANI and MND. Aβ40 levels above 186.31 pg/mL and NCAM-1 levels above 43.01 pg/mL distinguished MND from ANI (Figure 4B).

### 3.5. Additional nEV Protein Markers Examined Using Olink^®^ Proximity Extension Assay

**Figure 5:**
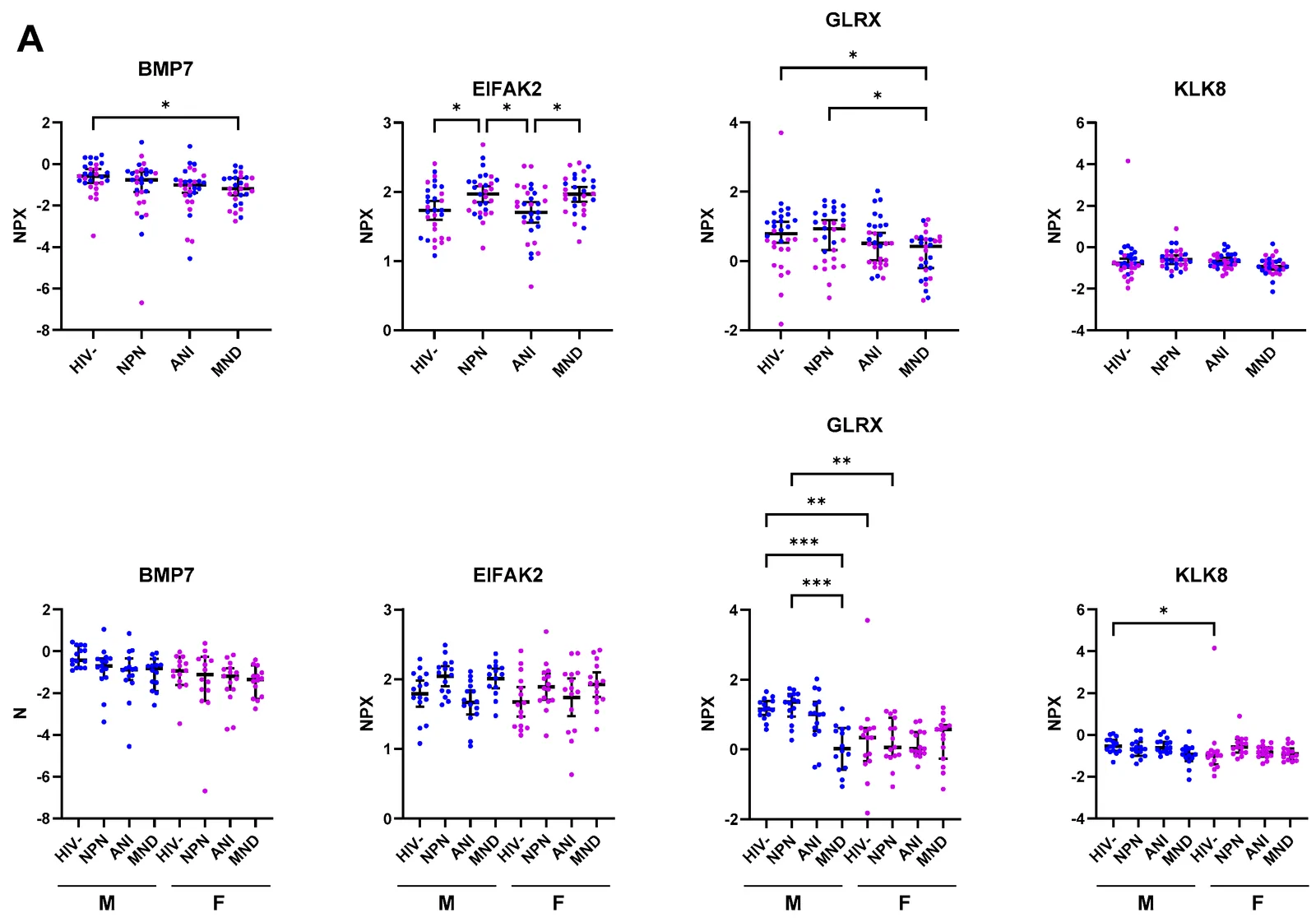

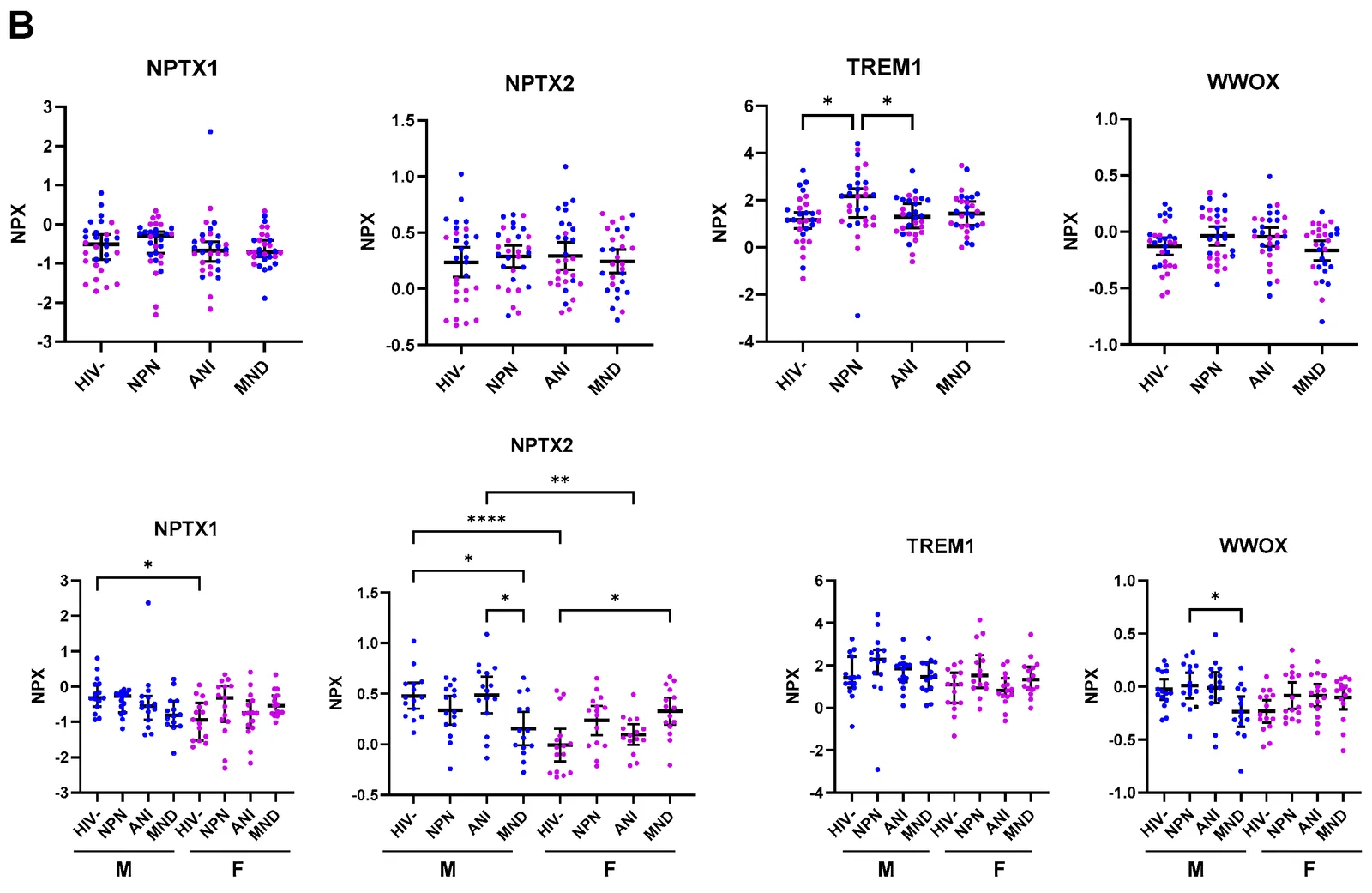
nEV 41-plex protein cargo (Olink^®^). (**A**,**B**) Top row: males and females combined into 4 cognition groups. Males are blue circles and females are pink circles. Bottom row: males (M) and females (F) separated into the 4 cognition groups, HIV−, NPN, ANI, and MND. The starting sample size for each group was *n* = 15. However, due to some samples being below assay sensitivities, the sample sizes analyzed here were as follows. In males, *n* = 14 for MND across all protein markers. In females for BMP7, *n* = 14 for HIV−, NPN, and MND. Horizontal bars indicate group medians with 95% confidence intervals as determined using Kruskal–Wallis tests, followed by post hoc Dunn tests for data that are not normally distributed (BMP7, GLRX, KLK8, NPTX1, TREM1). For data that are normally distributed (EIFAK2, NPTX2, WWOX), horizontal bars represent group means and 95% confidence intervals as determined by ordinary one-way ANOVA followed by Tukey tests to correct for multiple comparisons. Group differences are shown with asterisks defined as follows: * *p* ≤ 0.05, ** *p* ≤ 0.01, *** *p* ≤ 0.001, and **** *p* ≤ 0.0001.

**Figure A3:**
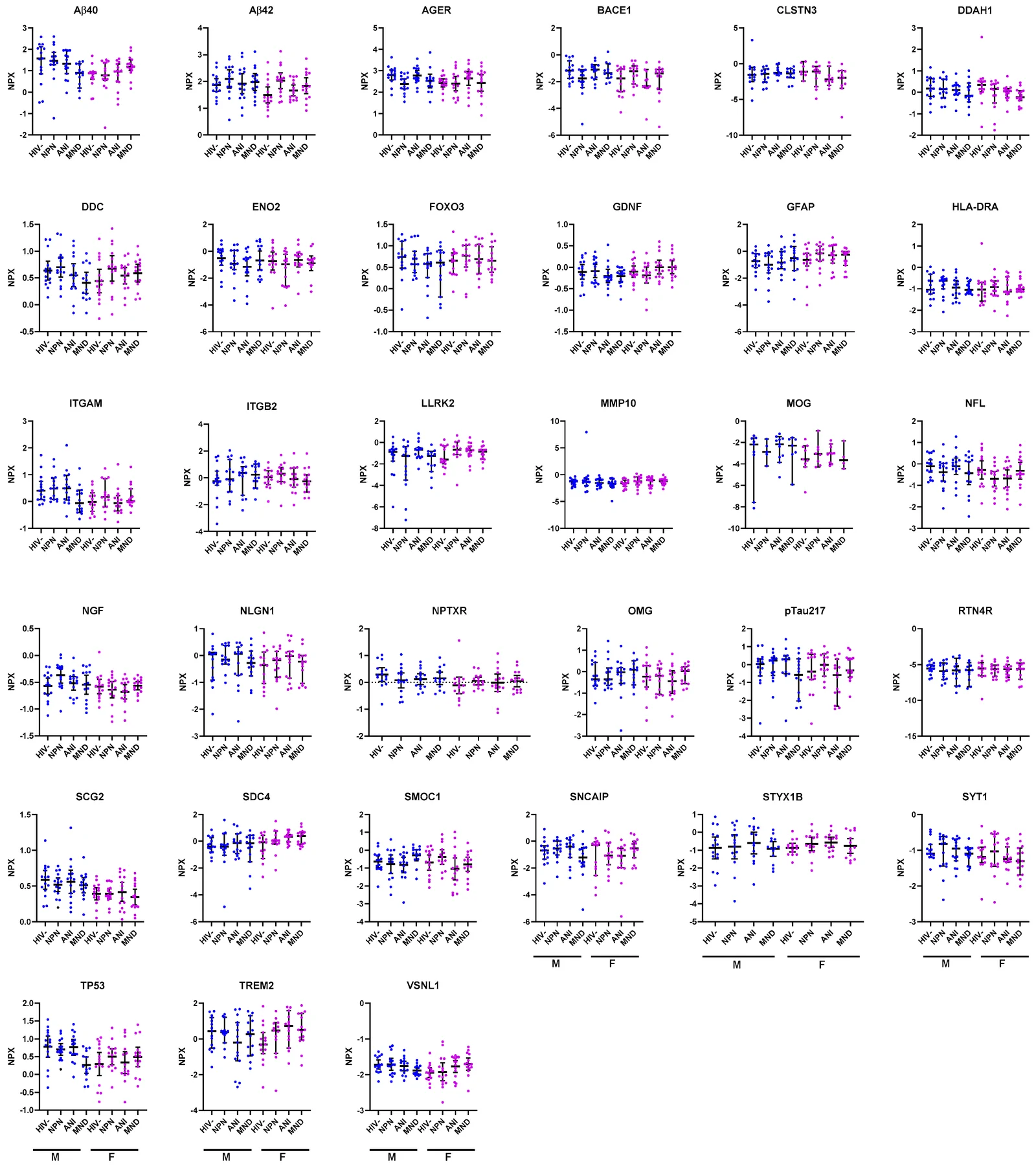
Thirty-three proteins examined by Olink^®^ proximity extension assay that did not show significant differences between the groups. Males (M) are illustrated as blue circles. Females (F) are illustrated as pink circles. Starting sample sizes for all groups were *n* = 15 except for male MND (*n* = 14). Due to assay sensitivity, some markers were not detectable for all of the input samples, resulting in reduced group sizes for analyses as follows: BACE1 (males 14 NPN, ANI, 12 MND; females 11 ANI), CLSTN3 (males: 13 HIV−, NPN, 10 ANI, 12 MND; females: 8 HIV−, 12 NPN, 10 ANI, 11 MND), ENO2 (females: 14 MND), LLRK2 (males: 13 MND; females: 12 NPN), MMP10 (males: 14 HIV−), MOG (males: 9 HIV−, 5 NPN, 11 ANI, 7 MND; females: 9 HIV−, 8 NPN, 10 ANI, 4 MND), pTau217 (males 13 NPN; females 14 NPN, ANI), RTN4R (males 14 NPN, ANI, 11 MND; females: 14 ANI, 13 HIV−, MND), SDC4 and SMOC1 (14 male NPN and female NPN), SNCAIP (14 female HIV−), STYX1B (14 male and 14 female MND), TREM2 (14 male NPN and female ANI, 13 female NPN). For all markers, protein levels were compared using Kruskal–Wallis tests followed by post hoc Dunn tests except Aβ42, AGER, DDC, GDNF, NFL, NGF, NPTXR, SCG2, SMOC1, STYX1B, TP53 and VSNL1. These 12 markers showed normal distribution of data and, thus, were analyzed by ordinary one-way ANOVA followed by Tukey tests to correct for multiple comparisons. Horizontal bars indicate group medians (Kruskal–Wallis tests) or group means (one-way ANOVA tests) with 95% confidence intervals.

**Figure A4:**
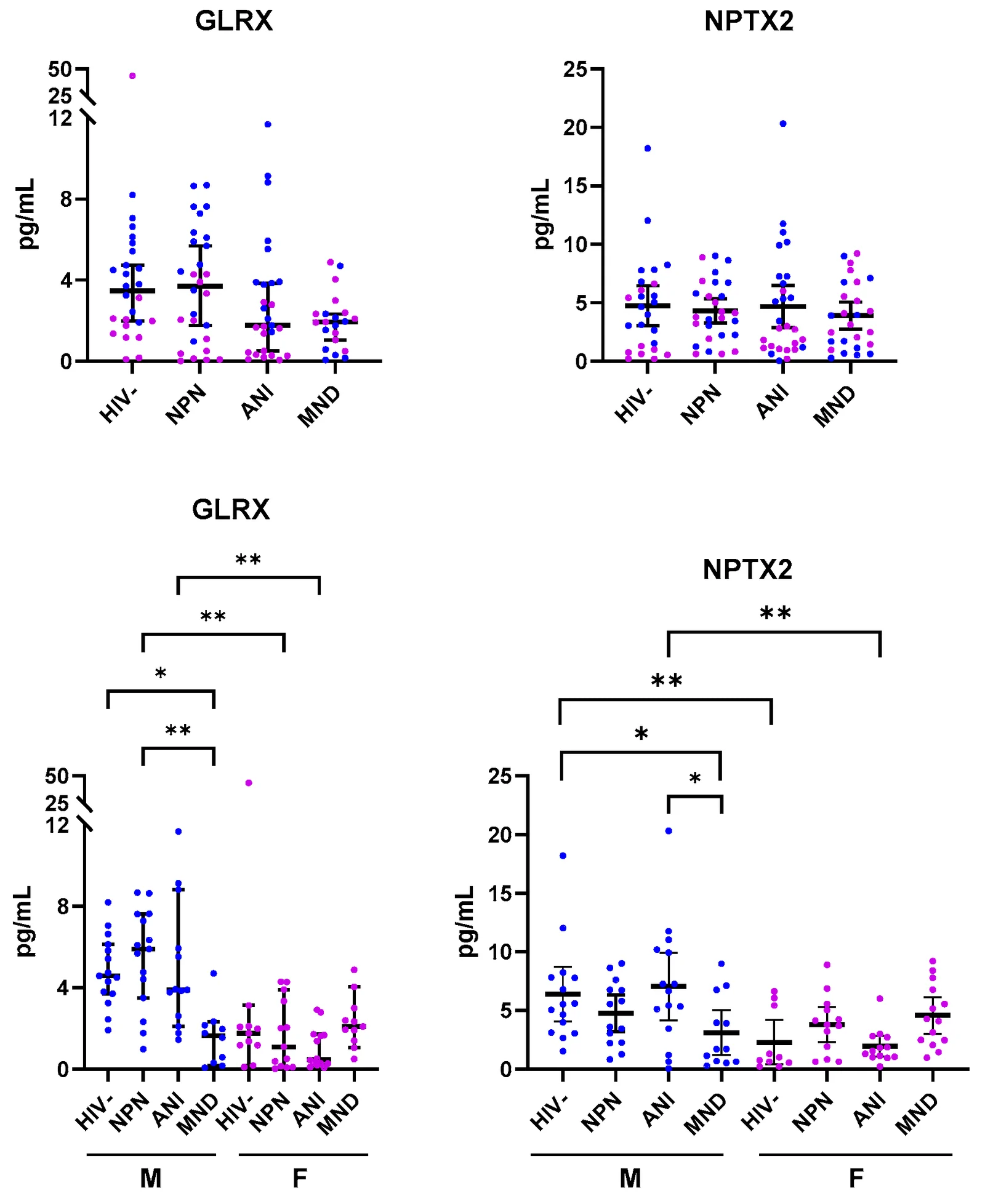
Quantitative levels of GLRX and NPTX2 in the HIV cognition groups. Top row: Males (M; blue circles) and females (F; pink circles) of the same cognition group combined. Bottom row: Males and females separated into their own groups. Due to sample limitations and assay sensitivity, group sizes shown here were as follows. For males: *n* = 15 (HIV−, NPN), *n* = 13 (ANI), *n* = 10 (MND). For females, *n* = 14 (ANI), *n* = 13 (NPN), *n* = 11 (HIV−, MND). Horizontal bars indicate group medians with 95% confidence intervals as determined using Kruskal–Wallis tests, followed by post hoc Dunn tests. Only significant group differences are shown with asterisks defined as follows: * *p* ≤ 0.05, and ** *p* ≤ 0.01.

Our first 2 panels using the Luminex format showed sex differences, with females having significantly different and possible diagnostic markers between cognition groups not seen in men. With the aim of finding potential diagnostic markers for men, we expanded the neurodegeneration protein pool by analyzing the nEVs using the Olink^®^ Target 48 Neurodegeneration multiplex proximity extension assay, which has 41 targets. Of the 41 proteins tested, 8 showed visible differences (Figure 5) while 33 showed little differences (Figure A3) between the sexes or sample groups. pTau 217 (phosphorylated tau at threonine 217), a new biomarker for Alzheimer’s disease (AD), was included in the panel and showed few differences between the sample groups in our cohort (Figure A3), whereas BMP7 (bone morphogenetic protein 7), EIFAK2 (interferon-induced double-stranded RNA-activated protein kinase), GLRX, and TREM1 (triggering receptor expressed on myeloid cells 1) did show group differences. In terms of sex differences, GLRX, KLK8 (kallikrein-related peptidase 8), NPTX1 (neuronal pentraxin-1), NPTX2 (neuronal pentraxin-2), and WWOX (WW domain-containing oxidoreductase) were higher in men than in women for the same cognition group.

GLRX was lower in the sex-combined ANI and MND groups compared to the sex-combined NPN and HIV− groups (Figure 5A). However, when the sexes were separated, it was the men driving these differences. As was seen in several other proteins, nEVs from MWoH had significantly higher levels of GLRX than WWoH (Figure 5, top row). MWH with MND had lower levels of GLRX compared to MWH with NPN or MWoH. There were also sex differences, as MWoH or MWH with NPN had significantly higher levels of GLRX than their female counterparts. In men, there appears to be a stepwise lowering of GLRX levels that associates with increasing cognitive impairment.

In our cohort, NPTX1 showed little difference in protein levels between sex-combined sample groups but did show a significant decrease in WWoH when compared to MWoH, with a pattern like KLK8. NPTX2 showed little difference between the 4 sex-combined groups but significant differences among the samples when the sexes were divided. MWH with MND had lower NPTX2 protein levels compared to MWoH or MWH with ANI. In contrast to the men, where there was a lowering of protein levels, WWH with MND had higher levels of NPTX2 protein compared to WWoH.

### 3.6. Discriminating Between the Cognition Groups in MWH

**Figure 6:**
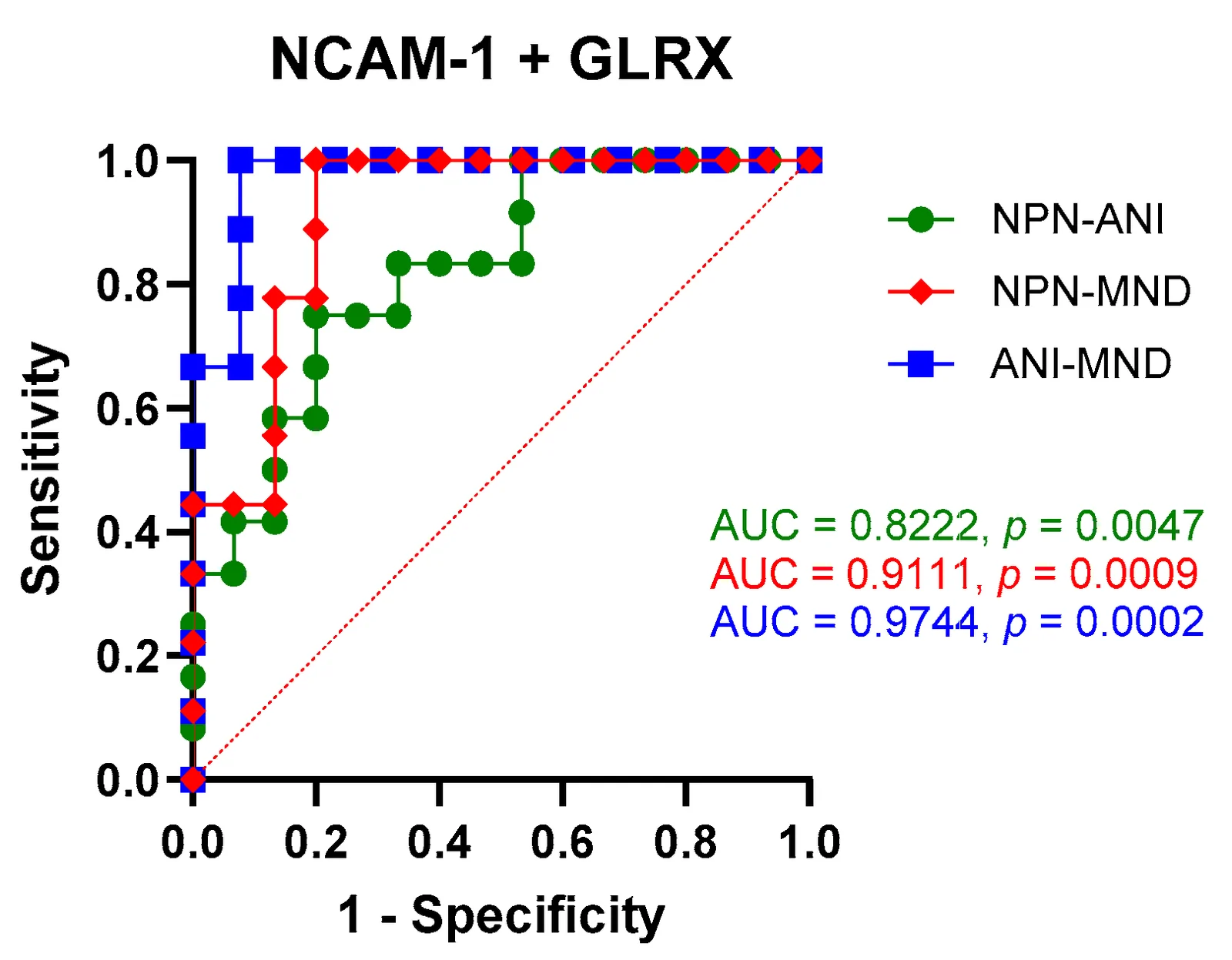
ROC (receiver operating characteristic) curve using dual predictors, NCAM-1 and GLRX, to discriminate between cognition groups in men. Quantitative values in pg/mL were used for both NCAM1 (Figure 2) and GLRX (Figure A4). The red dashed line represents the ROC curve for the classifier.

Since we were able to find single predictors that discriminated between the cognitive groups in WWH, but not in MWH, we performed multiple logistic regression and found that with GLRX plus NCAM-1, we were able to achieve AUCs above 0.80 (Figure 6 and Table A1). We also examined other protein marker additions to NCAM-1 but found the AUCs for the other combinations to be not as good (Table A1).

## 4. Discussion

Cognitive impairment in chronic well-controlled HIV continues to be an important clinical issue, with milder forms caused by multiple underlying conditions such as aging, cardiovascular conditions, ART toxicity, and mental health issues. In addition, there is strong evidence that there are sex differences in HIV cognitive profiles, with women showing more cognitive disadvantages [13,14,15] and weaknesses in different NP testing domains [15] than men. The molecular underpinnings of these sex differences are unclear. While it was not our intention to focus on men and women who are not infected with HIV, it is noteworthy that there were significant differences between the sexes in several nEV proteins. Specifically, NRGN, GLRX, NPTX2, pTau181, and TDP-43 were all higher in MWoH compared to WWoH, and these proteins are associated with synaptic dysfunction and neurodegeneration [19,20].

We previously reported, using a different cohort from the National NeuroAIDS Tissue Consortium (NNTC) with smaller sample sizes and a different set of biomarkers, that 14 proteins were differentially expressed among the HIV cognition groups with different patterns between the sexes [13]. The goal of this study was to find different fluid biomarkers, ones that are more specific to neurodegeneration, for each of the categories of HIV impairment, in particular ANI, since the symptoms at this stage may be more subtle than those seen in the early years of more severe HIV. Compared to CSF markers, plasma markers are a more rapid, less invasive, and inexpensive method to follow cognitive impairment at a time when treatment or cure is possible. Moreover, biological protein markers originating from neurons would also allow, in real time, insights into mechanistic changes, such as alterations in neuroinflammation or synaptic dysfunction that could progress to cognitive impairment.

An increasing number of studies are reporting the value and parallels of using nEVs instead of CSF as CNS markers of cognitive impairment [21,22]. One report assessed the concordance between plasma nEVs and CSF in AD [21]. In that multicenter study, there was excellent agreement for the markers Aβ42, tTau, and pTau181 in AD, amnestic mild cognitive impairment (aMCI), and control groups. Comparisons of the AUCs between nEVs and CSF showed no difference, suggesting that nEVs may substitute for CSF in biomarker analyses [21]. As these markers correlated with CSF, they also suggest agreement with brain pathological changes. We previously reported that nEVs from a small study of PWH and PWoH (22 men and 1 woman) and cognitive impairment had an increase in Aβ, NFL, and HMGB1 cargo [23]. We began to consider that chronic HIV may be a risk factor for AD. An epidemiology report showed that older PWH (especially women) have an increased incidence of AD and other dementias compared to people without HIV [24]. Both HIV and early AD have overlapping clinical manifestations, and it may be important to separate them as treatments for AD become available. While HIV does not necessarily progress to dementia, the early cognitive decline in older PWH may look like AD. Despite these earlier findings of parallels to AD, a recent study by us using a panel of 384 neurological proteins comparing the nEV cargo of PWH with PWoH with AD found no overlapping proteins [14,25]. In this report, we show for the first time the results for pTau217 inside nEVs isolated from plasma. pTau217 is a promising new biomarker for AD and has been shown to detect early pathological changes before clinical symptoms manifest [26,27,28]. pTau217 levels have been examined in plasma [29], CSF [29], and EVs derived from CSF [30]. nEVs are more representative of CSF cargo, but in a study looking at plasma and CSF pTau217 in AD, there were highly significant correlations [31,32]. We did not see differences in nEV pTau217 with sex or cognitive categories in the present study. Taken together, we do not believe that HAND is early AD.

HSP90 is a protein used to characterize EVs; it is also a molecular chaperone thought to be secreted during inflammation or stress, with low to undetectable levels under non-inflammatory conditions [33]. Since HSP90 is also involved in the proper folding of many cellular proteins needed for HIV-1 gene expression, it is thought to be a regulator of HIV latency, and inhibiting HSP90 blocks pathways to HIV reactivation [34]. Such a significant difference in HSP90 levels in neuronal nEVs between men and women may add a mechanistic variable for different approaches to treat men and women. In this study, we found nEV HSP90 protein levels to be higher in men than in women. This finding leads us to speculate that the latent virus reservoir in men may be less quiescent than in women living with HIV. This may explain the overall higher levels of protein expression in many of the markers examined in men (GAPDH, NFL, HMGB1, NRGN, pTau181, TDP43, KLK8, NPTX1, and NPTX2). This hypothesis is supported by a published prospective study of 26 well-matched pairs of ART-suppressed PWH reporting that HIV transcriptional activity in reservoir cells from men is higher than in women [35]. The reduced HSP90 in nEVs from women may be from hormonal changes such as menopause that may impact the HIV reservoir and reactivation [35]. In the present study, the mean age of women in all cognitive categories was in the low 50s, a year above the mean age of 49 for natural menopause [36,37].

Prior to this study, our initial hypothesis was that there would be a stepwise progressive difference between PWH with NPN and ANI and MND. That was not the case for most markers in general. Because this was a cross-sectional study, it is not possible to determine whether the observed biomarker patterns reflect disease progression, compensatory responses, or stable biological differences between groups. Nevertheless, we speculate that PWH in the NPN category may have mounted an effective response to HIV. This may involve an increase in nEV neurodegenerative proteins that are efficiently released from neurons into the periphery as a clearance mechanism to eliminate the toxic neuronal accumulation. Most of the nEV 10-plex toxic proteins showed a decrease in ANI from NPN. ANI may be the most common form of cognitive impairment in HIV and the most difficult to diagnose, as 16–19% of young healthy PWOH could be considered to have ANI [38]. Since ANI is asymptomatic and does not interfere with daily living, it is important to see if there is a neurological change in this group and the relevance and likelihood of it progressing to MND. The ANI group closely mimicked the group without HIV, showing lower levels of toxic proteins, suggesting an unresponsiveness in the cell as a result of chronic HIV. Although ANI does not always progress to MND, a longitudinal study suggested that it imparts a 2-6-fold increased risk [39]. Other factors contribute to progression, such as length of infection, age, and co-morbidities. Higher levels of nEV toxic proteins were observed among participants with symptomatic MND, similar to those seen in initial HIV; however, this may not be a beneficial mechanism for neuronal clearance but rather an impaired clearance process.

Divergent highs and lows observed with total Tau (tTau) in nEVs from MWH were not seen in WWH. tTau is a marker of neuronal injury and was increased in ANI compared to NPN and significantly decreased in MND, suggesting a different pathway in women. Likewise, the stepwise decrease in GLRX in men with HIV was not present in women with HIV and is a marker that could be followed over time in men. A decrease in GLRX is linked to an increase in oxidative stress and exacerbation of neurodegeneration in models of Parkinson’s disease (PD) [40] and increased neuronal death in postmortem PD brains [41].

Neuronal pentraxins (NPTX1 and NPTX2) are associated with synaptic activity, and low levels in CSF are used as biomarkers for cognitive dysfunction due to neurodegeneration [42] as seen in AD [32] and vascular dementia [43]. NPTX2 had a marked decrease in MWH with MND compared to MWH with ANI. There was also a sex difference, as NPTX2 was significantly decreased in WWH with ANI compared to MWH with ANI.

When we looked in women at the six proteins that are different between the three HIV cognition groups (Aβ40, Aβ42, FGF-21, KLK-6, NCAM-1, TDP43), we found that three of them (Aβ40, Aβ42 and TDP-43) form aberrant protein aggregates previously reported to be involved in cognitive impairment and neurological disease, with a fourth (KLK-6) having the capacity to generate amyloidogenic fragments by cleaving APP (amyloid precursor protein) [44]. These proteins have been implicated in AD, frontotemporal lobar degeneration, amyotrophic lateral sclerosis, Huntington’s disease, and others [45,46,47,48]. In men, we found the proteins that are differentially expressed between the three HIV cognition groups were NCAM-1, tTau, GLRX, NPTX2, and WWOX. Of these five differentially expressed proteins in MWH, two are involved in redox reactions (GLRX and WWOX), which directly influence a third protein (tTau). WWOX has been shown to bind JNK, ERK, and GSK-3β kinases, preventing hyperphosphorylation of tau by these kinases [49,50]. GLRX, as a glutathione-disulfide oxidoreductase, can modulate the redox state of tau, thereby reducing aberrant tau filament formation [51,52,53,54]. Compared to MWH with either NPN or ANI, we found MWH with MND had lower nEV protein levels of GLRX, WWOX, and tTau (Figure 2 and Figure 5). Although it did not reach statistical significance, there was also a trend towards lower levels of pTau181 (Figure 2) and pTau217 (Figure A3). Taken together, our findings support the hypothesis that in MWH and MND, lowered GLRX and WWOX protein levels may result in oxidative stress for the neuron accompanied by pathologic tau filament formation, resulting in reduced release of toxic tau proteins via nEVs.

Protein levels that varied amongst cognition groups were different between MWH and WWH in these early stages of asymptomatic or mild cognitive impairment. WWH had more pronounced protein levels that participate in pathological aggregation, while MWH had reduced protein levels that regulate redox and lower oxidative stress. We speculate that hormonal differences that began earlier in life may have laid the foundation for the subsequent molecular neurological changes that differ between the sexes. Published reports find that lower basal levels of autophagy in women are regulated by estrogen and reduced in postmenopausal women [55,56]. We found lower levels of HSP90 in women across all cognition categories (Figure 1). Together, these findings suggest that the combination of reduced molecular chaperone activity and autophagy sets women off on a path towards neurological decline that initially involves proteinopathies. These aberrant proteins may occur through misfolding and their subsequent lack of clearance from the cell. For men, the path towards neurological decline may initially involve a combination of diminished oxidoreductases [Figure 5] and pro-oxidative actions by testosterone [57,58] to create an environment of oxidative stress in the CNS. Although there may be divergent pathways because of hormonal sex differences, common mechanisms may also exist, such as that involving NCAM-1 (Figure 2), that applies to all PWH.

**Figure 7:**
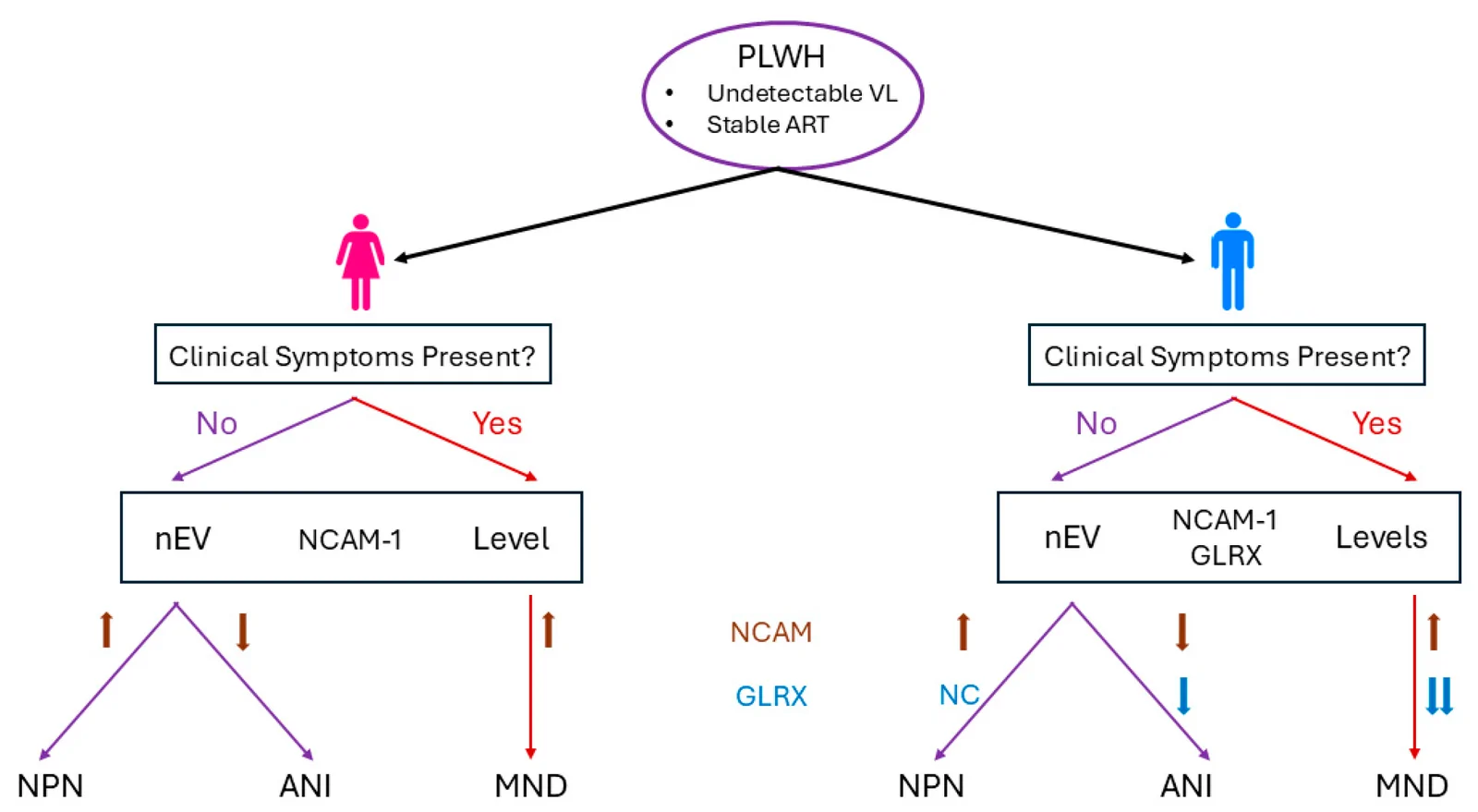
Hypothetical model for incorporating nEV protein markers into HAND diagnosis. NC, no change. Up arrow indicates an increase, and a down arrow indicates a decrease. One or two arrows are displayed to indicate lesser or greater magnitudes of change, respectively.

Based on the data presented in this study, we envision a conceptual model to include nEV proteins that would enhance diagnosis of neurocognitive categories of HIV and be more objective than NP testing (Figure 7). For example, using nEV NCAM-1 concentrations alone may differentiate NPN, ANI, and MND in women based on symptoms and a possible cutoff value of 43.01 pg/mL using our assay. To diagnose men, both NCAM-1 and GLRX may be used with a higher starting concentration of NCAM-1 (around 341 pg/mL in our assay) than in women, plus a stepwise decrease in GLRX from NPN to ANI to MND. Since this is a small, exploratory, and cross-sectional study, the diagnostic framework described in Figure 7 would need validation in larger independent cohorts, and longitudinal studies are required before specific nEV biomarker value cutoffs or clinical algorithms can be utilized.

Several studies show differences in cognition between men and women based on education and socioeconomic factors and suggest that cognitive dysfunction may not be caused by HIV infection [38,59]. Underdiagnosing cognitive impairment in HIV infection risks not being treated, and overdiagnosing can lead to stigma and unnecessary treatment. The benefit of having objective biological markers for cognitive impairment cannot be overstated.

There are limitations in this study. This was a cross-sectional observational study. We do not know how fluid the three cognitive groups are. PWH may stay at NPN or ANI for some time and may or may not progress to MND. The numbers are small when men and women are not grouped together, and there are four groups for each sex. Multiple nEV proteins were evaluated across several group comparisons, increasing the possibility of false-positive findings. Although multiple-comparison corrections were applied within individual group-comparison analyses, this exploratory study was not designed to provide confirmatory evidence for any single biomarker. Therefore, the findings should be interpreted as hypothesis-generating and should be evaluated in larger cohorts. The logistic regression and AUC analyses were intended to summarize marker discrimination within this exploratory dataset, and the small subgroup sizes limit the stability of resampling-based validation approaches such as cross-validation. Therefore, the observed discrimination should be interpreted cautiously and evaluated in larger cohorts before clinical diagnostic conclusions can be drawn. For all of these reasons, the findings in this study will need to be confirmed using larger, independent cohorts, as well as longitudinal data.

Other limitations include that the samples are from a biorepository where only small aliquots are available. The advantage of the MWCCS biorepository is the comprehensive epidemiological, clinical, and neuropsychological data that is available. The disadvantage is the challenge of performing numerous assays on small aliquots of plasma. The women in this cohort were mostly middle-aged (mean ages 51–54 years) and menopausal, suggesting that there may be divergent results within the cohort based on hormonal changes [60]. The progression of treated HIV concomitant with aging and comorbidity manifestations complicates conclusions and makes it difficult to put together meaningful cohorts. nEV isolation and characterization are still cumbersome, and standardization is necessary for clinical use. The evolving field of microfluidics may simplify isolation and targeting of specific proteins [25]. Another consideration for future studies might include disparities around race. Our previous study used different nEV proteins to compare men and women and cognitive impairment in a predominantly white cohort from the NNTC [13]. The present MWCCS cohort was predominantly black. While we did exclude people with pre-existing conditions to explain cognitive impairment, black men and women in the population have a higher rate of co-morbidities and dementia [61]. Future studies to validate the preliminary findings reported here should include a larger sample size, a wider age range, and be racially and ethnically diverse.

In the era of precision medicine, individual monitoring of brain diseases is possible. Changes in cognition could be followed with blood biomarkers such as nEVs as opposed to CSF sampling or neuropsychological testing and neuroimaging. The biologic sex difference should be considered as it may influence different treatment strategies. Blood biomarkers can take away inherent biases in treatment trials. In this study, we show that subtle cognitive differences such as ANI can be diagnosed without time-consuming or invasive testing.

## 5. Conclusions

Using plasma neuronal-enriched EVs, we found significant differences in protein cargo between men and women living with HIV and among cognitive groups: NPN, ANI, and MND. We posit that the mechanisms causing sex differences in HAND can be biologically differentiated and should be considered in diagnosis and treatment.

## Figures and Tables

**Table 1:** Demographics of sample groups consisting of HIV− healthy controls with normal cognition (HIV−), HIV+ with normal cognition (NPN), HIV+ with asymptomatic impaired cognition (ANI), and HIV+ with symptomatic mild impaired cognition (MND).

		HIV−		HIV+		
			NPN	ANI	MND	
		*n* = 31	*n* = 40	*n* = 30	*n* = 39	*p* Value
**Sex (%)**	Male	16 (52)	21 (52)	15 (50)	20 (51)	>0.9999 ^a^
	Female	15 (48)	19 (48)	15 (50)	19 (49)	
**Age in years, Range**	Group	46–62	44–66	46–65	45–64	
	Males	46–62	44–66	46–62	45–64	
	Females	46–61	45–62	46–65	45–61	
**Mean (SD)**	Group	53 (4)	52 (5)	53 (6)	51 (5)	0.2439 ^b^
	Males	53 (4)	52 (5)	53 (5)	51 (5)	0.7430 ^b^
	Females	53 (5)	52 (6)	54 (7)	51 (5)	
**ART in years, Range**	Group	NA	0.75–23.25	0–21.75	0–23.75	
	Males	NA	0.75–23.25	0–21.75	0–23.25	
	Females	NA	2.75–21.75	2.25–21.75	1.25–23.75	
**Mean (SD)**	Group	NA	10.3 (5.6)	10.2 (6.2)	10.7 (7.1)	0.9573 ^c^
	Males	NA	9.4 (5.7)	11.3 (5.2)	10.9 (6.7)	0.4060 ^b^
	Females	NA	11.4 (5.5)	9.1 (7.0)	10.4 (7.7)	
**Race (%)**	White group	6 (19.4)	4 (10.0)	4 (13.3)	4 (10.3)	0.6459 ^a^
	Black group	21 (67.7)	32 (80.0)	19 (63.3)	29 (74.4)	
	Other * group	4 (12.9)	4 (10.0)	7 (23.3)	6 (15.4)	
	White males	4 (25.0)	2 (9.5)	2 (13.3)	3 (15.0)	0.8848 ^a^
	Black males	11 (68.8)	17 (81.0)	11 (73.3)	16 (80.0)	
	Other * males	1 (6.2)	2 (9.5)	2 (13.3)	1 (5.0)	
	White females	2 (13.3)	2 (10.5)	2 (13.3)	1 (5.3)	0.6919 ^a^
	Black females	10 (66.7)	15 (78.9)	8 (53.3)	13 (68.4)	
	Other * females	3 (20.0)	2 (10.5)	5 (33.3)	5 (26.3)	
**Ethnicity (%)**	Hispanic group	6 (19.4)	3 (7.5)	4 (13.3)	5 (12.8)	0.5226 ^a^
	Non-Hispanic group	25 (80.6)	37 (92.5)	26 (86.7)	34 (87.2)	
	Hispanic males	3 (18.8)	2 (9.5)	2 (13.3)	2 (10.0)	0.8652 ^a^
	Non-Hispanic males	13 (81.2)	19 (90.5)	13 (86.7)	18 (90.0)	
	Hispanic females	3 (20.0)	1 (5.3)	2 (13.3)	3 (15.8)	0.6326 ^a^
	Non-Hispanic females	12 (80.0)	18 (94.7)	13 (86.7)	16 (84.2)	
**Education in years**	<High school group	4 (12.9)	3 (7.5)	1 (3.3)	3 (7.7)	0.3919 ^a^
	H.S. Grad./Some College group	15 (48.4)	28 (70.0)	22 (73.3)	28 (71.8)	
	College Grad. or higher group	12 (38.7)	9 (22.5)	7 (23.3)	8 (20.5)	
	<High school males	1 (6.2)	1 (4.8)	1 (6.7)	1 (5.0)	0.9480 ^a^
	H.S. Grad./Some college males	8 (50.0)	13 (61.9)	9 (60.0)	14 (70.0)	
	College Grad. or higher males	7 (43.8)	7 (33.3)	5 (33.3)	5 (25.0)	
	<High school females	3 (20.0)	2 (10.5)	0 (0.0)	2 (10.5)	0.2669 ^a^
	H.S. Grad./Some college females	7 (46.7)	15 (78.9)	13 (86.7)	14 (73.7)	
	College Grad. or higher females	5 (33.3)	2 (10.5)	2 (13.3)	3 (15.8)	
**HIV viral load**	All were undetectable at <20 copies/mL					

SD, standard deviation. NA, not applicable. ART, antiretroviral therapy. H.S., high school. Grad., graduate. * Other races include Asians, native Hawaiians/Pacific Islanders, native Alaskans/American Indians, multi-racial, and other races not specifically listed. ^a^ Fisher’s exact test. ^b^ One-way ANOVA with Tukey correction for multiple comparisons. ^c^ Kruskal–Wallis test followed by Dunn’s post-test. Sample groups by HIV status and cognition is shown in black. Males are shown in blue. Females are shown in pink.

**Table 2:** Logistic regression using single predictors for NPN, ANI, and MND.

**NPN and ANI Discrimination**											
***Males***						***Females***					
**Predictor**	**AUC**	***p* Value**	**Overall % Correctly Classified**	**β1 Odds Ratio**	**95% CI**	**Predictor**	**AUC**	***p* Value**	**Overall % Correctly Classified**	**β1 Odds Ratio**	**95% CI**
NCAM-1	0.8603	0.0003	77.78%	0.9924	0.9858 to 0.9969	Aβ42	N/A	N/A	N/A	N/A	N/A
Aβ40	0.8317	0.0008	83.33%	0.9958	0.9919 to 0.9982	NCAM-1	N/A	N/A	N/A	N/A	N/A
Aβ42	0.7825	0.0043	72.22%	0.5683	0.3531 to 0.8206	FGF21	0.9963	<0.0001	93.94	0.7345	0.4639 to 0.8792
tTau	0.7810	0.0045	58.33%	1.002	0.9985 to 1.007	KLK6	0.9926	<0.0001	93.94	0.6145	0.2557 to 0.8523
KLK6	0.7429	0.0141	61.11%	0.9678	0.9378 to 0.9917	Aβ40	0.9648	<0.0001	90.91	0.9836	0.9583 to 0.9959
Counts	0.6603	0.1051	72.22%	1	1.000 to 1.000	TDP43	0.9407	<0.0001	93.94	0.9979	0.9961 to 0.9991
TDP43	0.6413	0.1533	66.67%	1	1.000 to 1.001	NRGN	0.8519	0.0006	72.73	0.9465	0.9042 to 0.9782
pTau181	0.6159	0.2415	72.22%	1.07	0.9987 to 1.183	pTau181	0.8111	0.0024	69.70	0.6917	0.4605 to 0.9213
HMGB1	0.6159	0.2415	66.67%	1	1.000 to 1.000	HMGB1	0.7778	0.0067	69.70	0.9991	0.9983 to 0.9998
Age	0.5973	0.2828	54.76%	1.059	0.9357 to 1.210	Mode Size	0.6649	0.1031	58.82	1.04	1.001 to 1.091
NRGN	0.5968	0.3277	58.33%	0.9967	0.9871 to 1.006	Median Size	0.6246	0.2182	52.94	1.043	0.9936 to 1.107
FGF21	0.5968	0.3277	55.56%	0.9855	0.9580 to 1.008	Mean Size	0.6211	0.2315	55.88	1.043	0.9865 to 1.113
CD81	0.5683	0.4903	61.11%	3.786E+66	3.364e-029 to 3.011e+189	ART	0.6136	0.2287	69.23	0.9448	0.8444 to 1.050
Mode Size	0.5651	0.5107	58.33%	1.019	0.9642 to 1.080	HSP90	0.5947	0.3490	55.88	0.9884	0.9549 to 1.019
NFL	0.5571	0.5636	58.33%	0.9987	0.9860 to 1.010	GAPDH	0.5842	0.4052	55.88	0.9981	0.9926 to 1.003
Median Size	0.5524	0.5965	58.33%	1.017	0.9314 to 1.113	NFL	0.5807	0.4250	55.88	1.005	0.9527 to 1.060
GAPDH	0.5508	0.6077	55.56%	1	0.9992 to 1.002	αSyn (total)	0.5807	0.4250	52.94	1.003	0.9912 to 1.017
CD9	0.5492	0.6189	61.11%	2,501,004	1.329e-008 to 1.066e+023	Counts	0.5614	0.5439	64.71	1	1.000 to 1.000
ART	0.5490	0.5920	56.10%	1.036	0.9302 to 1.161	CD81	0.5439	0.6646	55.88	6.3E-13	2.193e-193 to 1.535e+142
HSP90	0.5397	0.6884	63.89%	1.002	0.9970 to 1.008	CD63	0.5368	0.7157	55.88	1.4E-124	0.000 to +infinity
αSyn (total)	0.5365	0.7121	58.33%	1	0.9948 to 1.006	CD9	0.5158	0.8760	55.88	2.45E-12	1.610e-083 to 4.165e+050
Mean Size	0.5238	0.8098	58.33%	1.01	0.9173 to 1.115	tTau	0.5148	0.8850	45.45	0.003925	−0.004897 to 0.01453
CD63	0.5016	0.9872	58.33%	135,018	7.222e-142 to 2.154e+139	Age	0.5120	0.8986	61.54	1.023	0.9190 to 1.140
**ANI and MND Discrimination**											
***Males***						***Females***					
**Predictor**	**AUC**	***p*** **Value**	**Overall % Correctly Classified**	**β1 Odds Ratio**	**95% CI**	**Predictor**	**AUC**	***p*** **Value**	**Overall % Correctly Classified**	**β1 Odds Ratio**	**95% CI**
tTau	0.9474	<0.0001	82.35	0.9592	0.9240 to 0.9814	Aβ40	N/A	N/A	N/A	N/A	N/A
Aβ40	0.9333	<0.0001	91.18	1.011	1.004 to 1.024	NCAM-1	N/A	N/A	N/A	N/A	N/A
NCAM-1	0.8772	0.0002	73.53	1.009	1.004 to 1.016	FGF21	0.9825	<0.0001	94.12	1.203	1.081 to 1.469
Aβ42	0.7860	0.0047	70.59	1.839	1.190 to 3.287	TDP43	0.9404	<0.0001	91.18	1.002	1.001 to 1.004
HMGB1	0.7298	0.0231	64.71	0.9995	0.9990 to 0.9999	KLK6	0.9474	<0.0001	88.24	1.229	1.096 to 1.495
TDP43	0.7018	0.0461	70.59	0.9993	0.9985 to 0.9998	Aβ42	0.9404	<0.0001	88.24	860.7	6.994 to 1,005,951,790
KLK6	0.6982	0.0500	67.65	1.03	1.000 to 1.068	NRGN	0.7825	0.0052	64.71	1.048	1.012 to 1.100
Age	0.6654	0.0738	55.00	0.9038	0.7824 to 1.027	Mode Size	0.7737	0.0068	73.53	0.9302	0.8693 to 0.9774
pTau181	0.6561	0.1227	61.76	0.9338	0.8477 to 1.000	pTau181	0.7474	0.0145	64.71	1.243	1.021 to 1.794
CD81	0.6167	0.2433	71.43	2.25E-213	0.000 to 1.643e-030	HMGB1	0.7333	0.0211	64.71	1.001	1.000 to 1.002
Mean Size	0.6000	0.3173	57.14	0.9641	0.8585 to 1.070	Median Size	0.6930	0.0564	64.75	0.9365	0.8704 to 0.9906
αSyn	0.5933	0.3506	60.00	0.9974	0.9907 to 1.004	Mean Size	0.6702	0.0925	64.71	0.9398	0.8711 to 0.9991
Mode Size	0.5733	0.4634	62.86	0.9752	0.9212 to 1.028	NFL	0.6333	0.1875	55.88	0.9892	0.9423 to 1.034
Median Size	0.5700	0.4839	62.86	0.9573	0.8674 to 1.046	αSyn	0.6263	0.2118	61.75	0.9921	0.9775 to 1.005
GAPDH	0.5683	0.4944	54.29	0.9996	0.9983 to 1.001	Counts	0.5860	0.3955	55.88	1	1.000 to 1.000
NFL	0.5467	0.6407	54.29	0.9973	0.9849 to 1.009	HSP90	0.5544	0.5908	44.12	1.001	0.9778 to 1.027
FGF21	0.5333	0.7418	55.88	1.007	0.9825 to 1.036	ART	0.5476	0.6177	52.63	1.025	0.9394 to 1.124
CD9	0.5200	0.8415	57.14	0.01329	6.972e-016 to 213,432,915,476	CD81	0.5351	0.7187	55.88	1.92E+22	2.025e-129 to 2.076e+204
CD63	0.5150	0.8808	60.00	7.88E-109	0.000 to 2.682e+103	tTau	0.5263	0.7948	41.18	0.9989	0.9905 to 1.007
HSP90	0.5150	0.8808	57.14	0.999	0.9941 to 1.004	CD63	0.5123	0.9034	55.88	3.75E-52	0.000 to +infinity
ART	0.5075	0.9352	52.50	1.007	0.9110 to 1.114	CD9	0.5070	0.9447	52.94	5.67E-10	1.882e-090 to 1.924e+071
NRGN	0.5053	0.9585	44.12	0.9981	0.9882 to 1.008	Age	0.5042	0.9649	60.53	0.975	0.8710 to 1.089
Counts	0.5000	>0.9999	52.50	1.105	0.5932 to 2.073	GAPDH	0.5018	0.9862	55.88	1	0.9948 to 1.006

N/A = Not available due to inability to perform a logistic regression fit because of perfect or near-perfect separation.

## Data Availability

The raw data supporting the conclusions of this article will be made available by the authors on request.

## Abbreviations

The following abbreviations are used in this manuscript:

Aβ40	Amyloid beta 1–40
Aβ42	Amyloid beta 1–2
AGER	Advanced glycosylation end product-specific receptor
ANI	Asymptomatic Neuropsychologically Impaired PWH
αSyn	Alpha synuclein (total)
BACE1	Beta-secretase 1
BMP7	Bone morphogenetic protein 7
CLSTN3	Calsytenin-3
CNS	Central nervous system
CSF	Cerebral spinal fluid
DDAH1	N(G),N(G)-dimethylarginine dimethylaminohydrolase 1
DDC	Aromatic-L-amino-acid decarboxylase
FGF-21	Fibroblast growth factor 21
FOXO3	Forkhead box protein 03
EIF2AK2	Interferon-induced, double-stranded RNA-activated protein kinase
ENO2	Gamma-enolase
GAPDH	Glyceraldehyde 3-phosphate dehydrogenase
GDNF	Glial cell line-derived neurotrophic factor
GFAP	Glial fibrillary acidic protein
GLRX	Glutaredoxin-1
HIV−	Controls without HIV and with normal cognition
HLA-DRA	HLA class II histocompatibility antigen, DR alpha chain
HMGB1	High mobility group box 1
HSP90	Heat shock protein 90
ITGAM	Integrin alpha-M
ITGB2	Integrin beta-2
KLK-6	Kallikrein-related peptidase 6
KLK-8	Kallikrein-related peptidase 8
LRRK2	Leucine-rich repeat serine/threonine-protein kinase 2
MMP10	Stromelysin
MND	Mild Neurological Disorder in PWH
MOG	Myelin-oligodendrocyte glycoprotein
MWH	Men with HIV on stable ART with undetectable viral load
MWoH	Men without HIV
NCAM-1	Neural cell adhesion molecule 1
nEV	Neuron-enriched extracellular vesicle
NFL	Neurofilament light polypeptide
NGF	Beta-nerve growth factor
NLGN1	Neuroligin-1
NP	Neuropsychological
NPN	Neuropsychologically normal PWH
NPTX1	Neuronal pentraxin-1
NPTX2	Neuronal pentraxin-2
NPTXR	Neuronal pentraxin receptor
NRGN	Neurogranin
OMG	Oligodendrocyte-myelin glycoprotein
pTau181	Microtubule-associated protein tau phosphorylated at residue 181
pTau217	Microtubule-associated protein tau phosphorylated at threonine 217
PWH	Participants/people with HIV on stable ART with undetectable viral load
PWoH	Participants/people without HIV
RTN4R	Reticulon-4 receptor
SCG2	Secretogranin-2
SDC4	Syndecan-4
SMOC1	SPARC-related modular calcium-binding protein
SNCAIP	Synphilin-1
STX1B	Syntaxin-1B
SYT1	Synaptotagmin-1
TDP-43	TAR DNA binding protein 43
TP53	Cellular tumor antigen p53
TREM1	Triggering receptor expressed on myeloid cells 1
TREM2	Triggering receptor expressed on myeloid cells 2
VSNL1	Visinin-like-protein 1
WWH	Women with HIV on stable ART with undetectable viral load
WWoH	Women without HIV
WWOX	WW domain-containing oxidoreductase (WWOX)

## Appendix D

**Table A1:** Multiple logistic regression using main effects model for NCAM-1 plus one other protein to discriminate between cognition groups in males.

Discrimination Between Groups *(NCAM-1 and GLRX)*	AUC	*p* Value	Overall % Correctly Classified	Predictor	β Odds Ratio	95% CI	AICc
NPN and ANI	0.8308	0.0030	75.00	NCAM-1 (pg/mL)	0.9924	0.9842 to 0.9975	33.7
				GLRX (pg/mL)	0.8443	0.5739 to 1.197	
NPN and MND	0.9111	0.0009	83.33	NCAM-1 (pg/mL)	1.002	0.9979 to 1.006	24.06
				GLRX (pg/mL)	0.3937	0.1361 to 0.7006	
ANI and MND	0.9744	0.0002	90.91	NCAM-1 (pg/mL)	1.011	1.003 to 1.033	15.41
				GLRX (pg/mL)	0.09826	0.002283 to 0.5335	
**Discrimination Between Groups *(NCAM-1 and NPTX2)***	**AUC**	***p*** **Value**	**Overall % Correctly Classified**	**Predictor**	**β Odds Ratio**	**95% CI**	**AICc**
NPN and ANI	0.8381	0.0019	72.41	NCAM-1 (pg/mL)	0.9940	0.9873 to 0.9983	35.54
				NPTX2 (pg/mL)	1.113	0.8752 to 1.483	
NPN and MND	0.7381	0.0396	73.08	NCAM-1 (pg/mL)	1.001	0.9980 to 1.003	38.99
				NPTX2 (pg/mL)	0.7436	0.5136 to 1.002	
ANI and MND	0.9000	0.0004	85.19	NCAM-1 (pg/mL)	1.007	1.002 to 1.016	27.41
				NPTX2 (pg/mL)	0.7581	0.5047 to 1.006	
**Discrimination Between Groups *(NCAM-1 and WWOX)***	**AUC**	***p*** **Value**	**Overall % Correctly Classified**	**Predictor**	**β Odds Ratio**	**95% CI**	**AICc**
NPN and ANI	0.8267	0.0023	73.33	NCAM-1 (pg/mL)	0.9932	0.9864 to 0.9976	36.53
				WWOX (NPX)	0.6398	0.009586 to 29.55	
NPN and MND	0.7590	0.02	64.29	NCAM-1 (pg/mL)	1.001	0.9978 to 1.003	38.60
				WWOX (NPX)	0.009336	7.202e-005 to 0.3401	
ANI and MND	0.8769	0.0007	75.00	NCAM-1 (pg/mL)	1.008	1.002 to 1.016	30.19
				WWOX (NPX)	0.05838	0.0004880 to 3.045	

CI, confidence interval. AICc, Akaike’s corrected information criterion.
